# Soft, all-in-one, nanomembrane wearable system for advancing neonatal health monitoring in Ethiopia

**DOI:** 10.1038/s41746-025-01974-8

**Published:** 2025-09-25

**Authors:** Lauren Zhou, Michele Joseph, Yoon Jae Lee, Diva Yadav, Likhit Nayak, Julia Woodall, Jared Matthews, Ira Soltis, Firehiwot Markos Mekuria, Kullehe Haddis Yeshanew, Yonas Kebede Mamo, Abebaw Fekadu, Asrat Demtse, Rudolph Gleason, Woon-Hong Yeo

**Affiliations:** 1 George W. Woodruff School of Mechanical Engineering, College of Engineering, Georgia Institute of Technology, Atlanta, GA USA; 2 Wearable Intelligent Systems and Healthcare Center (WISH Center) at the Institute for Matter and Systems, Georgia Institute of Technology, Atlanta, GA USA; 3 Center of Biomedical Engineering, Addis Ababa Institute of Technology, Addis Ababa University, Addis Ababa, Ethiopia; 4 Center for Innovative Drug Development and Therapeutic Trials for Africa College of Health Sciences, Addis Ababa University, Addis Ababa, Ethiopia; 5 School of Electrical and Computer Engineering, Georgia Institute of Technology, Atlanta, GA USA; 6 Department of Computer Science, Georgia State University, Atlanta, GA USA; 7 Tikur Anbessa Specialized Hospital, Addis Ababa University, Addis Ababa, Ethiopia; 8 Neonatal Intensive Care Unit (NICU), Department of Pediatrics and Child Health, College of Health Sciences, Addis Ababa University, Addis Ababa, Ethiopia; 9 Department of Global Health & Infection, Brighton and Sussex Medical School, Brighton, United Kingdom; 10 Wallace H. Coulter Department of Biomedical Engineering, College of Engineering, Georgia Tech and Emory University School of Medicine, Atlanta, GA USA; 11 Korea KIAT-Georgia Tech Semiconductor Electronics Center (K-GTSEC) at the Institute for Matter and Systems, Georgia Institute of Technology, Atlanta, GA USA

**Keywords:** Engineering, Electrical and electronic engineering

## Abstract

The neonatal period is critical and stressful, particularly in low-resource settings where existing monitoring methods for newborns are sporadic and labor-intensive. There is a pressing need for an affordable, user-friendly device that offers continuous and effective monitoring. Here, we present a soft, all-in-one, wearable system designed for continuous wireless monitoring of neonatal health in low-resource settings. Developed using advanced packaging technologies, this system features a chest-mounted patch and a forehead-mounted pulse oximeter that transmit real-time data to a smartphone app. The integrated device measures clinical-grade parameters, including electrocardiograms, heart rate, respiration rate, temperature, and blood oxygen saturation, showing excellent correlation with hospital-grade tabletop devices and reduced motion artifacts. This pilot study demonstrates a significant improvement over current monitoring methods by providing continuous oversight, ensures critical events are not missed, and automates the process for enhanced neonatal care, resulting in 84% satisfaction by parents interested in using the system at home.

## Introduction

In 2022, 2.3 million children died globally during the neonatal period (0–28 days), which represented 47% of all under-five child mortality^1^. Nearly half (1.1 million) of these neonatal deaths occurred in sub-Saharan Africa; the neonatal mortality in sub-Saharan Africa remains the highest globally, with a rate of 27 deaths per 1000 live births in 2022 (https://www.who.int/news-room/fact-sheets/detail/newborn-mortality). In Ethiopia, significant efforts have led to a reduction in child mortality rates by 4.9 deaths per 1000 live births annually since 2005. However, neonatal mortality has declined by only 0.6 deaths per 1000 live births per year during the same period^1^. Consequently, the proportion of under-five deaths occurring during the neonatal period increased from 32% in 2005 to 55% in 2019, with 74% of these neonatal deaths happening within the first seven days of life^2^.

**Fig. 1:**
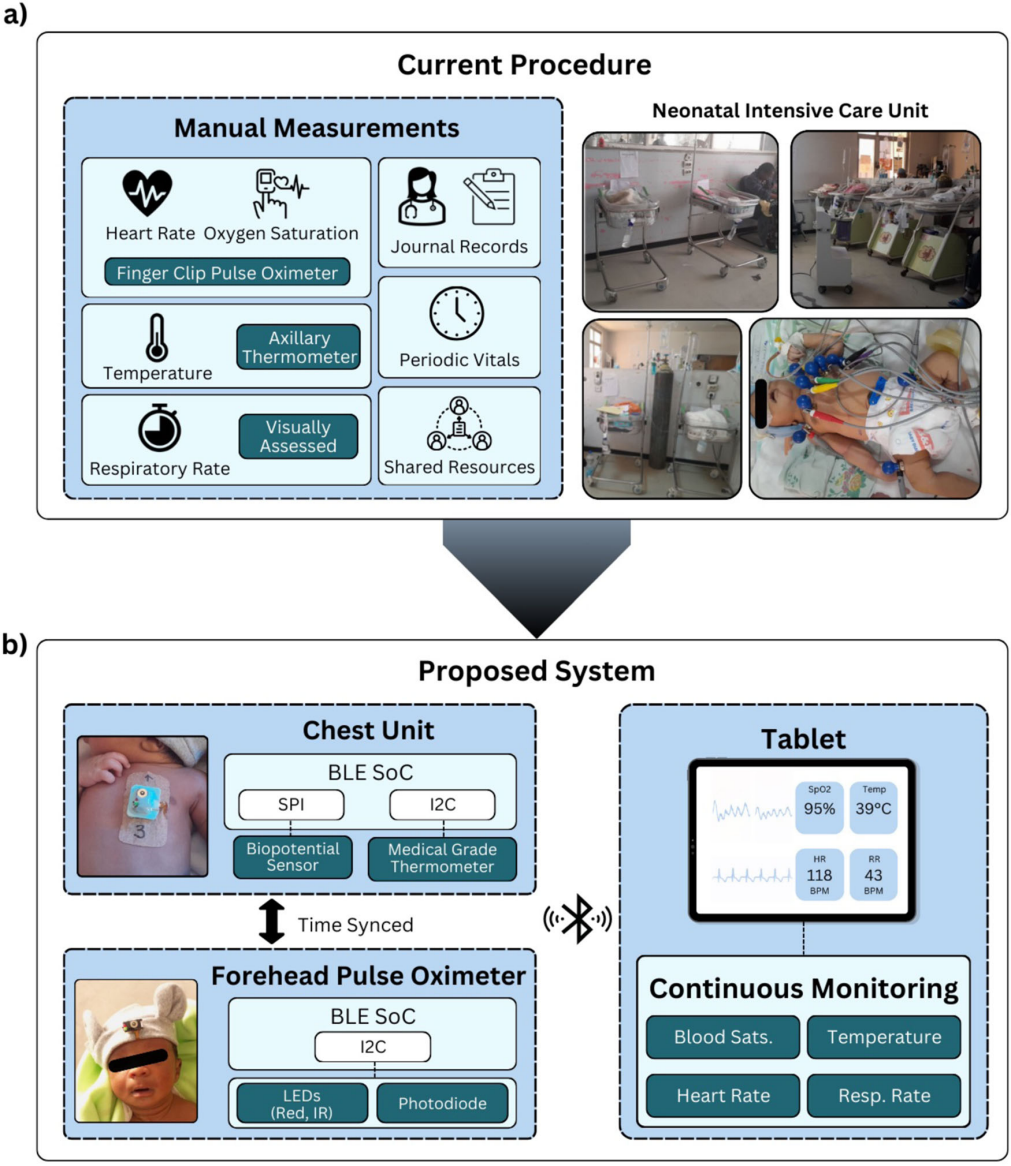
Overview of a soft, all-in-one, nanomembrane wearable bioelectronic system to improve neonatal monitoring in Ethiopia. **a** Details of the current procedure that relies on manual, intermittent measurements for heart rate, respiration rate, blood oxygen, and temperature measurements. **b** Visualization of the proposed wearable and wireless system for continuous vital monitoring. A biopotential sensor measures ECG, and a medical-grade thermometer measures skin temperature at the chest. The forehead-mounted pulse oximeter is easily attached to a headband with Velcro and measures PPG from the forehead. Both devices are time-synced and communicate wirelessly via BLE to a custom mobile app. The Flutter framework allows compatibility across a wide range of consumer products, including smartphones, tablets, and computers, maximizing applicability with users’ pre-existing devices. The mobile app features an intuitive interface with high-contrast visual elements and real-time alerts for vital sign abnormalities. Due to limited and unpredictable internet connectivity, the app computes offline heart rate, respiration rate, oxygen saturation, and temperature, and saves all recorded data locally until it can be saved to the cloud for long-term storage for later reference. The app processes data compression and local storage optimization to operate reliably in non-connected multi-day environments, with automatic data synchronization when internet connectivity becomes available.

At Tikur Anbessa Specialized Hospital (TASH) in Addis Ababa, a large public specialized referral hospital, the neonatal mortality rate stood at 29.7% between 2013 and 2018 (https://www.unicef.org/ethiopia/media/391/file/Child%20Survival%20Strategy%20in%20Ethiopia%20.pdf). The leading causes of death in these neonates were infections, prematurity, asphyxia, and congenital malformations. A study conducted at TASH’s Neonatal Intensive Care Unit (NICU) from July 2011 to June 2012 further revealed that out of 3277 admitted neonates, 855 (26%) were preterm. Among these preterm infants, only 69.3% survived to discharge. Hypothermia was a major contributing factor to mortality, with 95.9% of deceased preterm infants presenting with admission temperatures below 36.5 °C^3^. Key challenges contributing to these deaths include a shortage of trained neonatal care nurses, low nurse-to-patient ratios (ranging from 1:5 to 1:8), insufficient equipment, inadequate resources for infection prevention, and the absence of continuous monitoring of vital signs, all of which result in delays in identifying critical care needs for neonates. Currently, neonatal monitoring at TASH relies on thermometers, pulse oximeters, and manual counting of heart and respiratory rates (Fig. 1a). These methods require multiple devices and are performed intermittently with measurements logged in handwritten journals. Because healthcare workers must manually record these measurements, this approach increases clutter in the NICU, depends heavily on nurse availability, disturbs the neonates due to handling, and causes discomfort from the placement of multiple sensors. The lack of continuous monitoring often leads to delayed detection of life-threatening conditions such as hypothermia, respiratory distress, and apnea.

The Federal Democratic Republic of Ethiopia’s Ministry of Health has committed to ending preventable newborn and child deaths by 2035. This mission aligns with the United Nations Sustain Sustainable Development Goal 3.2 of reducing neonatal mortality to at least 12 per 1000 live births and under-five mortality to at least 25 per 1000 live births by 2030 (https://www.who.int/data/gho/data/themes/topics/sdg-target-3_2-newborn-and-child-mortality). Achieving these targets requires innovative, cost-effective technologies that enable continuous monitoring of high-risk neonates, ensuring early detection of critical health conditions and timely intervention to reduce neonatal mortality.

Here, this study reports the feasibility, accuracy, usability, and acceptability of the wearable system for continuous monitoring of neonates in an Ethiopian NICU setting. While previous works^4–6^ have developed similar wearable systems for neonates (Table 1), our study in developing wearable devices prioritizes low manufacturing cost and improved device longevity for reduced reliance on consumables and expensive component replacement. The proposed system (Fig. 1b) is a dual-mounted, wireless, flexible, and smart device that continuously tracks and displays heart rate, respiratory rate, blood oxygen saturation (SpO2), and temperature on a smartphone or a tablet with an embedded alert system, which can significantly enhance the effectiveness of neonatal care and reduce the discomfort associated with current practices. In addition to its advanced functionality, the system is biocompatible, thermally safe, and minimizes skin irritation, posing minimal risk to participants. Insights from parent feedback and the acceptability and usability questionnaires are also analyzed to assess the potential of the proposed system for clinical use in Ethiopian healthcare settings.

**Table 1:** Comparison of the characteristics of recently developed wearable health monitoring systems for neonates

Reference	Signal	Data transmission	Sensor placement	Device form factor	Reuseable	Replaceable components	Cost	Use case
This work (2025)	Heart Rate Respiration Temperature SpO2	Bluetooth, Mobile App	Chest + Forehead	Flexible wireless adhesive chest patch + non-adhesive oximeter	X	X	<$60	NICU monitoring in low resource countries
^4^ (2025)	Heart Rate Respiration Temperature SpO2 Body Posture	Bluetooth, Mobile App	Chest + Forehead	Flexible wireless adhesive chest patch + non-adhesive oximeter	X		<$140	At home monitoring for infants with single ventricle heart disease
^5^ (2020)	Heart Rate Respiration Temperature SpO2 Body Posture	Bluetooth, Mobile App	Chest + Hand/foot	Flexible wireless adhesive chest patch + adhesive oximeter	X			Standard clinical care in NICUs
^6^ (2019)	Heart Rate Respiration Temperature SpO2	NFC, Mobile App	Chest + Hand/foot	Flexible wireless adhesive chest patch + adhesive oximeter				Standard clinical care in NICUs

## Results

### Design and structure of the chest and forehead devices

**Fig. 2:**
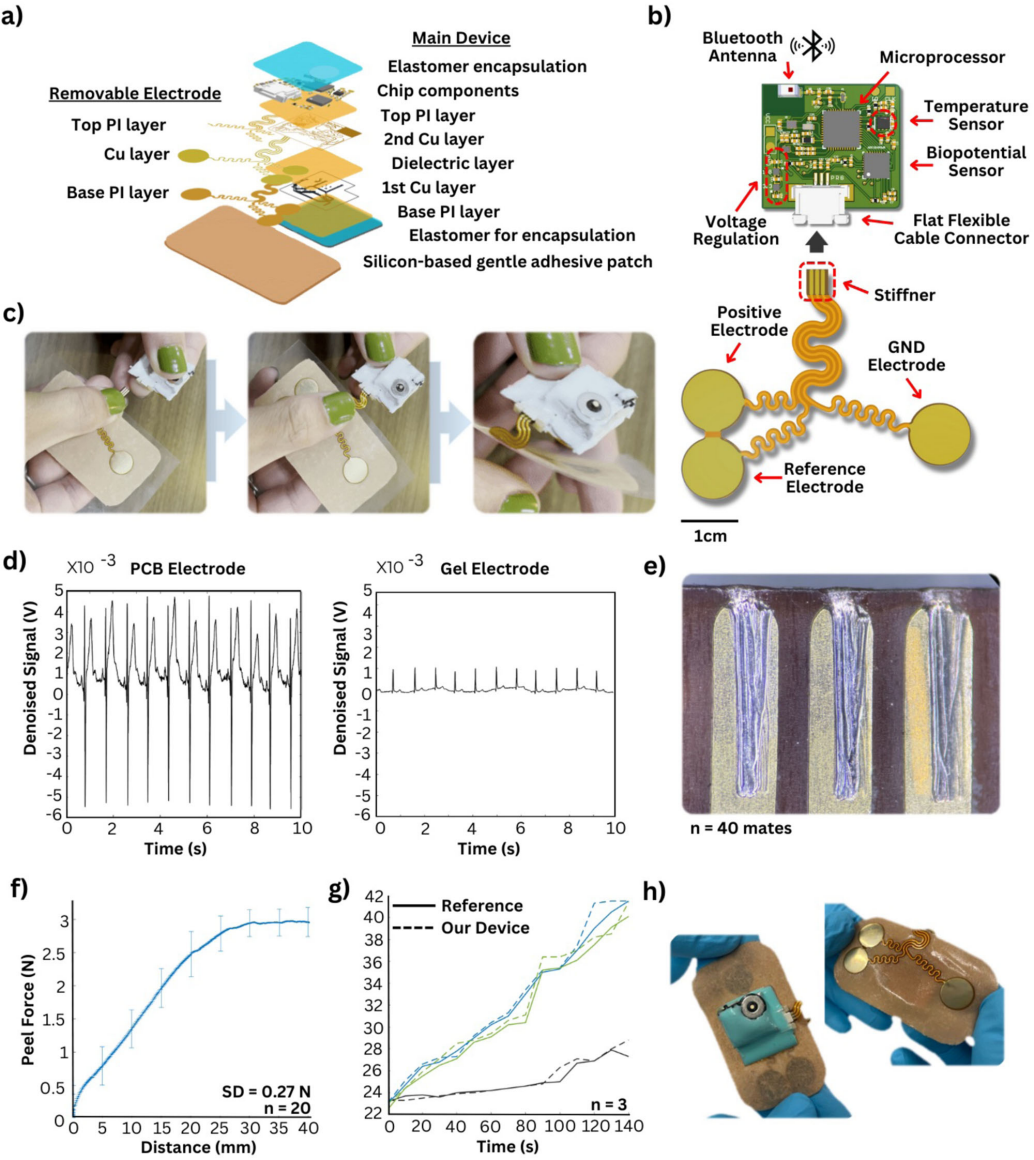
Mechanical design and characterization of the chest patch. **a** Exploded view of the device, including a wireless device, stretchable electrodes, soft encapsulation, and a biocompatible elastomer-based adhesive patch. **b** Circuit design for cardiovascular monitoring. **c** Incremental demonstration of removal and replaceable printed circuit board (PCB) electrodes using a flat flexible cable connector. **d** Signal performance comparison between PCB electrodes and traditional gel electrodes. **e** Degradation of the PCB electrodes over 40 mating cycles at the pressure-based connection point, showing the failure point of maximum matings for a single PCB electrode before needing replacement. **f** Peel test showing the integrity of reusable elastomer-based adhesive even after 20 cycles of isopropyl alcohol-based sanitization. **g** Temperature tracking performance of our device compared to a heat source with a low stabilization period from a 23 °C to 43 °C range, with high accuracy, showing accuracy across the operation range for health monitoring. **h** Photo of a fully encapsulated patch.

Figure 2a shows an exploded view of the complete chest device. Consisting of two flexible circuit boards, the primary processing module houses the electrical circuitry and chip components, completely enveloped between layers of water-tight elastomer encapsulation. The flexible circuit board consists of two copper signal layers separated by polyimide (PI) insulating substrates for low-noise, high-resolution applications. The reusable and replaceable dry electrodes are a single-layer printed circuit board with large, exposed pads serving as the electrode contact points. The electrode board only has one conductive copper layer stacked between two PI layers, with a small stiffening layer on the connecting junction to add rigidity and mating reusability. It is pre-attached to an adhesive patch that is coated with a thin layer of high-tack biocompatible elastomer-based adhesive that is gentle for neonatal skin. Figure 2b details the key designs and functional components of the cardiovascular monitoring patch, including processors, sensors, antenna, interconnects, power management, communication, and electrodes. The total power consumption of the device is approximately 5 mW when paired to Bluetooth and has an idle consumption of 2 mW. The device is powered by a rechargeable 70 mAh lithium-polymer (LiPo) battery, allowing 14 h of continuous use when fully charged. As demonstrated in Fig. 2c, the stiffened portion of the electrode PCB is simply inserted into the flat flexible cable connector on the main board, and the electrodes are folded beneath themselves, allowing a small 8.9 cm by 3.8 cm footprint. Traditional Ag/AgCl electrodes used in hospital settings are single-use and require conductive gels that irritate the skin and reduce signal quality over time. In the TASH NICU, reusable suction cup electrodes ($100) are used. However, nurses complain that the probes either fail to work or are unavailable, resorting to manual counting with an auscultation or clip oximeter. The PCB electrode utilizes the dry electrode sensing mechanism to allow long-term reusability with simple sanitization, eliminating the need for skin preparation, and is extremely low cost. In a study comparing the signal quality of our replaceable PCB electrodes to conventional gel electrodes on unprepped skin (Fig. 2d), the PCB electrodes registered an SNR of 25.35 dB, outperforming gel electrodes with an SNR of 12.06 dB on a clinical-grade reference device (BioRadio). While measured simultaneously (experimental setup shown in Fig. S1), the plotted data are not synchronized due to being recorded from two separate Bluetooth devices. The higher T-waves on the PCB electrode waveform are due to the placement of the electrodes on adult physiology, where the electrodes are directly over the heart rather than spanning the heart; on neonatal physiology, this amplification is not observed. An impedance density comparison further validated quantitative differences, where the dry gold PCB electrode exhibited an impedance density of 1.2 kΩ/cm² (impedance 1 kΩ, area 81 mm²), compared to 0.064 kΩ/cm² (impedance 0.145 kΩ, area 225 mm²) for conventional gel electrodes, as detailed in Fig. S2. To determine the device’s longevity and to identify the weak point for the removable electrodes, several sets of electrodes were inserted and removed from the FFC connector, and the resistance of each electrode from the sensing pad to the corresponding electrical pad on the PCB was measured. While the resistance for each of the three electrodes ranged slightly from 0-0.1 Ohms until finally creating an open circuit, the maximum number of mating cycles between a single electrode PCB and an FFC connector is limited to 30 cycles, and replacement electrodes are $1.95 (estimated from the cost of 120 PCBs for $124.34 from a 3rd party manufacturer). After examining possible failure points, Fig. 2e shows a microscopic view of the connecting pads of the electrodes. While the FFC connector can withstand at least 5 full electrodes of 30 mating cycles, the pressure-based electrical connection would flatten and remove conductive material from the PCB, eventually creating an open circuit. Another weak point that affects the longevity of this device is the adhesive of the reusable bandage. Using an elastomer-based adhesive rather than an acrylate- or rosin-based adhesive, the device adheres via Van der Waals forces that do not form singular permanent bonds to the skin and remain gentle for fragile infant skin. However, debris, dead skin, and dust may adhere to the device over time with use, reducing adhesion. Therefore, a peel test with isopropyl alcohol sanitization was conducted. Figure 2f shows the results of the peel test, demonstrating maintained adhesion force with repeated applications and sanitizations for 20 cycles. Figure 2g summarizes the temperature tracking performance of the device temperature sensor compared to a heat source with a low stabilization period from a 23 °C to 43 °C range with high accuracy, showing accuracy across the operation range for health monitoring: the experimental setup of this test appears in Fig. S3. The devices are thermally safe, with negligible heat generation from the devices (Fig. S4). Figure 2h shows the fully functional version of the integrated device with an encapsulated processing module and electrodes.

**Fig. 3:**
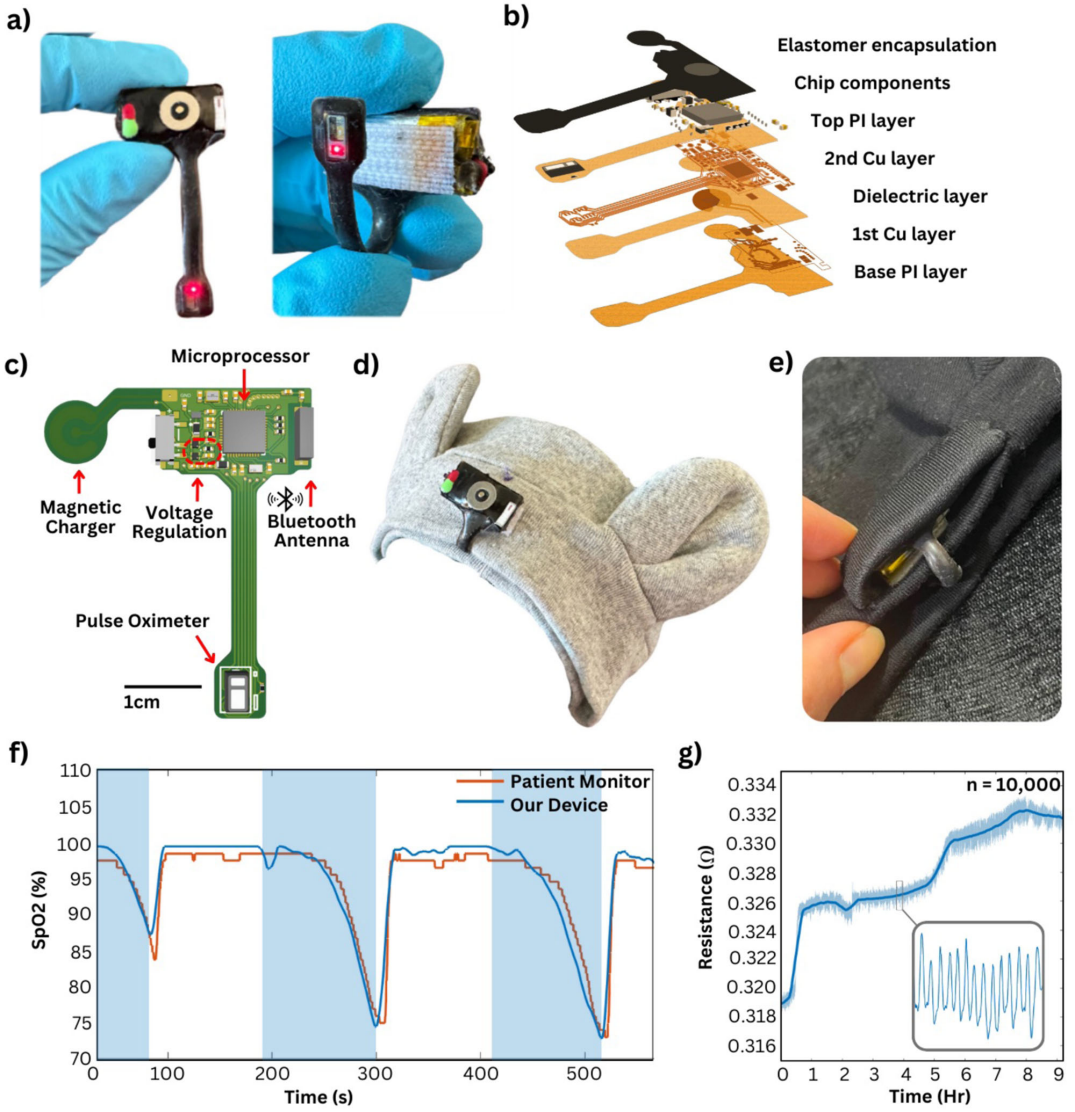
Mechanical design and characterization of the forehead pulse oximeter. **a** Detailed view of the device, with a flexible portion that bends and attaches with Velcro. **b** Exploded view of the wireless device. **c** Circuit design for forehead-mounted pulse oximeter. **d** Mounting headband. **e** An optional cover for the device for parents who are wary of the device’s electrical appearance. **f** Demonstration of measurement agreement of our device compared to a commercial patient monitor, where the shaded regions indicate breath hold for desaturation. **g** Measured ground plane resistance changes of our device with a 180° bending test (*n* = 10,000), capturing negligible changes.

Figure 3a showcases the wireless pulse oximeter sensor that measures blood oxygenation from the forehead. The bulk of the device is mounted atop its rigid powering battery with a flexible extension housing the PPG sensor at the tip. The device adheres to a wide neonatal headband with Velcro, allowing long-term reusability, and the sensor is simply tucked beneath the headband to lie flush on the center of the forehead. The forehead is an ideal blood oxygen measurement location for its stability and high vascular density^7^. Typical NICU blood oxygen monitoring is conducted on the hand or foot, which is subject to motion artifacts due to uncontrollable infant limb movement, making it difficult to attain accurate measurements. Because of the nature of how infants lie on their backs, the motion of their head is limited while the headband stays on securely, reducing motion artifacts. In addition, for infants who experience hypothermia or low body temperatures, which is common for infants in the Ethiopian NICU, distal measurement locations like the hand and foot often face limited perfusion to protect vital organs^8–11^. Therefore, for a measurement that relies on high perfusion for accuracy, the forehead is an excellent measurement location. The forehead-mounted device shows great stability in data recording even when walking and running (Figure S5), thus garnering reliable readings on a stable patient. As an all-in-one device, the composing layers of the device are identical to the functional chest module, with alternative copper signal and insulating PI layers (Fig. 3b). The device is coated with an elastomer encapsulation (Ecoflex 00-30, Smooth-On) to protect the electrical components and improve the durability of the device. The height of the PPG sensor is 1.55 mm, which protrudes out from the PCB. It also has relatively sharp corners that irritate the skin; therefore, an extra layer of elastomer is poured around the sensor to reduce the sensor imprint, making the device more comfortable for long-term wear. Figure 3c shows the functional blocks of the device, including the antenna and microprocessor, power regulation, and magnetic charging mechanism. The device shares the same communication core (Nordic Semiconductors, nRF52832) and voltage regulation circuit (ABLIC, S-1318) as the chest module. After complete component assembly, the magnetic charger portion of the flexible PCB folds over and is epoxied atop the components for a strong hold. The device is powered with a 110 mAh LiPo battery and has a power consumption rate of 5 mW when connected to Bluetooth and 2 mW when idle, allowing 22 h of use from a full charge. The pulse oximeter sensor is an off-the-shelf component (MAXIM, MAX30102) that functions with 660 nm and 940 nm wavelengths of light. Figure 3d shows the application of the proposed pulse oximeter to a neonatal headband, while Fig. 3e captures an optional modification that can be used to cover the device for parents wary of the device’s appearance. The device’s sensing spectrum was validated in comparison to a finger clip reference device on *n* = 15 healthy adults during a breath hold test. Figure 3f summarizes the comparison of measured SpO2 between the forehead-mounted and reference devices, ranging between 73% and 100%. To observe the electrical robustness of the flexible circuit with repeated bending, a 180° bend test (Fig. S6) of *n* = 10,000 was conducted with only a 14.34 milliohms increase in ground plane resistance, with the results shown in Fig. 3g.

### Real-time measurement study in the neonatal intensive care unit

**Fig. 4:**
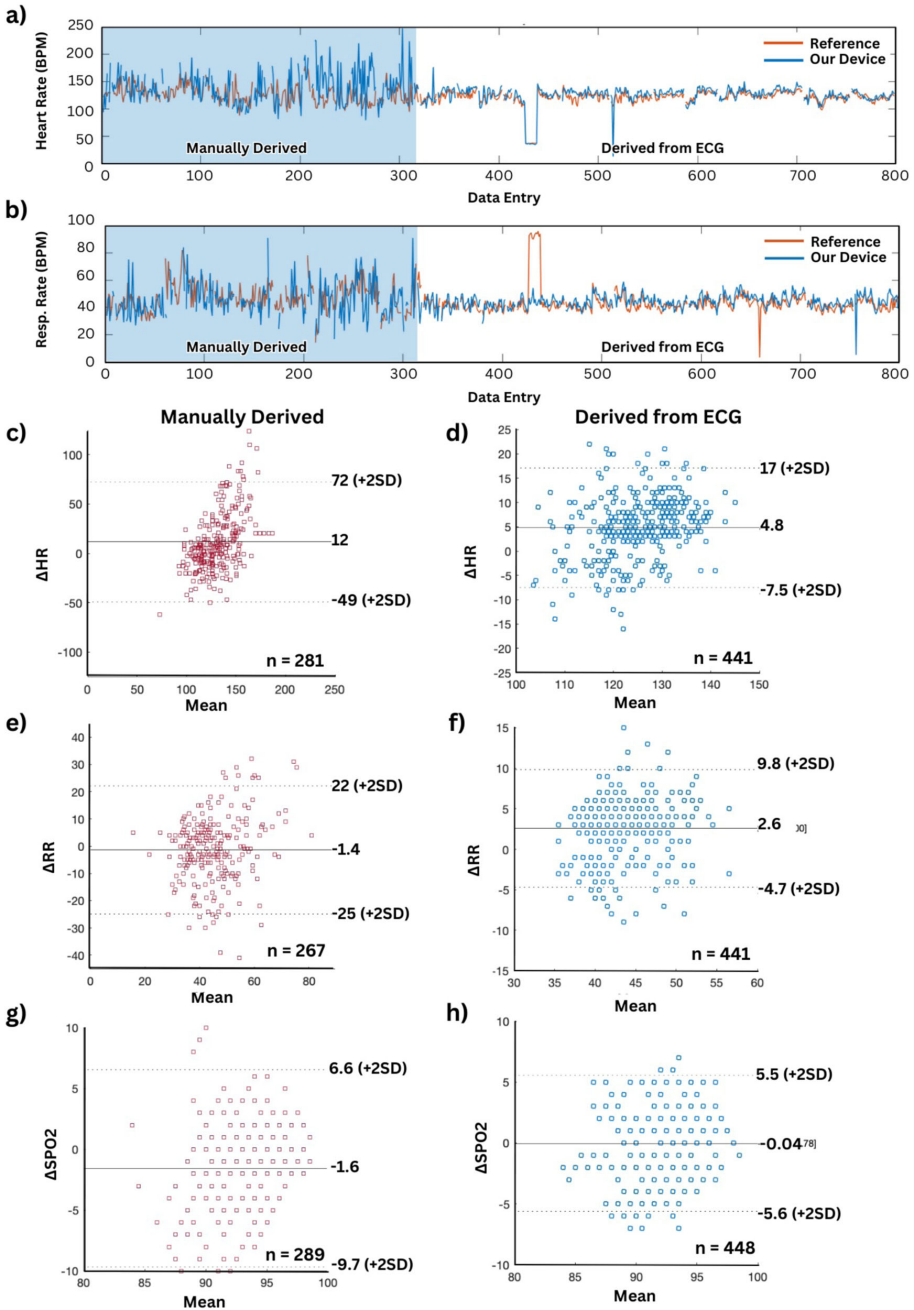
Data analysis and real-time measurement validation study. **a** Comparison of recorded heart rate data entries where the shaded regions indicate manually derived values with a pulse oximeter clip. **b** Comparison of recorded respiration rate data entries, where the shaded regions indicate manually derived values by counting over a 60 s duration. **c** Bland-Altman analysis for heart rate comparison between our device and manually collected reference for 10 observed neonatal patients (*n* = 281). **d** Bland-Altman analysis for heart rate comparisons between our device and ECG reference for 15 observed neonatal patients (*n* = 441). **e** Bland-Altman analysis for respiration rate comparisons between our device and manually collected reference for 10 observed neonatal patients (*n* = 267). **f** Bland-Altman analysis for respiration rate comparisons between our device and ECG reference for 15 observed neonatal patients (*n* = 441). **g** Bland-Altman analysis for SpO2 calculations between our device and manually collected reference for 10 observed neonatal patients (*n* = 289). **h** Bland-Altman analysis for SpO2 comparisons between our device and reference for 15 observed neonatal patients (*n* = 448).

To evaluate the feasibility of the proposed device system in Ethiopia, a pilot study with 25 neonates was conducted to assess the first generation of the device, with additional clinical studies with large sample sizes following this paper. Nurses made their rounds collecting vital signs from each patient using traditional monitoring protocols once per hour and recording the values in a paper journal, with an example of the digitized logs in Table S1. Our system was worn continuously, and nurses would write the values shown on the tablet each time they collected vitals manually. For the first 10 patients, reference heart rate and blood oxygen are measured using auscultation and a finger clip-style wireless sensor (Fig. S7a, b). Reference respiration rate is manually counted by a nurse or clinician in a 60 s observation. Reference temperature readings were compared to a digital axillary thermometer (Fig. S7c). After examining 10 patients using traditional methods, 15 patients had reusable suction cup electrodes attached, which were used to monitor ECG-derived heart rate and respiration rate (Fig. S7d). The ECG patient monitor was connected to a screen without electronic data saving capabilities. Thus, nurses logged the displayed values in a written journal alongside the values reported by our system. The change in the quality of the collected readings is distinguishable in Fig. 4a, b, where “Data Entry” is the individual logs collected by the nurses, and the values in the shaded region are from the manually derived portion of the study. Logged values that were out of normal range for neonates (HR < 100 BPM, RR < 34 BPM | RR > 60 BPM, SpO2 > 100%) were removed, with complete data shown in Fig. S8. Due to the handling required for auscultation and to attach a clip-style pulse oximeter that is pinched onto the hand of neonates for heart rate measurements, the comparative values in the shaded region show great discrepancies between our system and the manual reference. The values stabilize greatly when compared to patients using an ECG-based reference, likely due to continuous monitoring of ECG-based vital signs determination, reducing perturbation of the patients during rounds. Figure 4c–h show the statistical analysis of the manually derived (red) and ECG-derived (blue) reference values compared to those collected from our system. Manually derived heart rate and respiration rate show a mean difference of 12 beats per minute with a 95% confidence interval (CI) of [−49, 72] and a mean difference of −1.4 breaths per minute with a 95% CI of [−25, 22], respectively. ECG-derived heart rate and respiration rate showed much improved results with a mean difference of 4.8 beats per minute with a 95% confidence interval (CI) of [−7.5, 17] and a mean difference of 2.6 breaths per minute with a 95% CI of [−4.7, 9.8], respectively. While they show large deviations in individual data entries, these results are acceptable considering neonatal respiratory sinus arrhythmia, which refers to the normal variation in heart rate that directly corresponds to respiration^12^. For neonates, heart rate varies rapidly by 10–20 beats per minute or more, increasing with inhalation and decreasing with exhalation^13^. Considering the data collection method with a paper journal and rapid breathing rate of neonates, the results align with this phenomenon. Moderate levels of agreement between the derived peripheral capillary oxygen saturations from our forehead pulse oximeter and reference device, with a mean difference of −1.6% and 95% CI if [−9.7, 6.6] during the manual portion of the study and −0.04% and 95% CI of [−5.6, 5.5] during the ECG portion of the study. Considering the lack of a continuous measurement reference, it is difficult to assess the accuracy of the comparison between our system and the TASH NICU reference method.

**Fig. 5:**
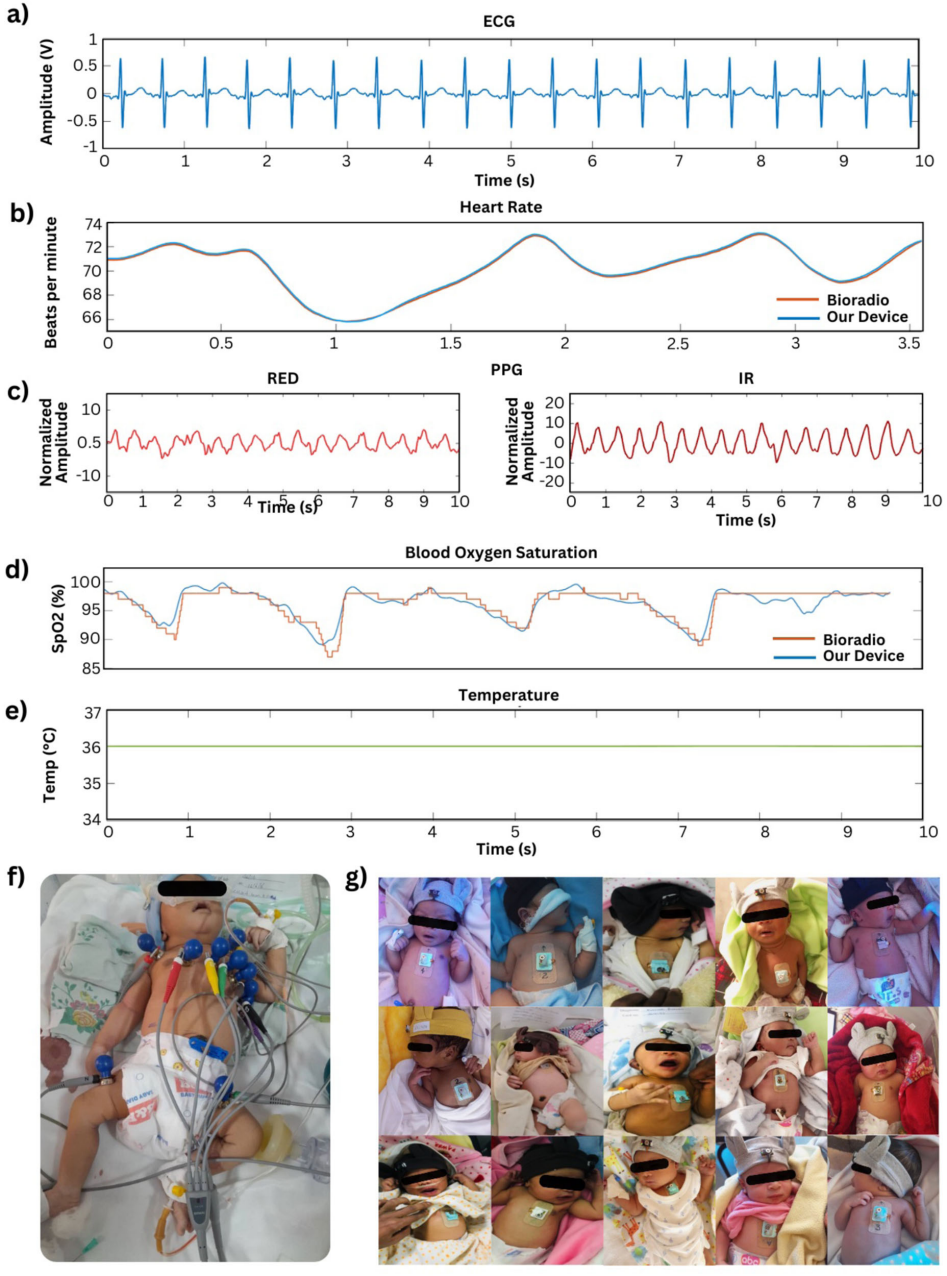
System validation and acceptability in the NICU. **a** 10 s segment of filtered ECG data from a neonate in the study. **b** Derived heart rate values between our device and a known reference (BioRadio) on a healthy adult. **c** 10 s segment of filtered PPG data from a neonate in the study. **d** Results of the breath hold test on a healthy adult in comparison with a reference device. **e** 10 s segment of temperature sensor data from a neonate in the study. **f** Neonate undergoing traditional ECG-based continuous monitoring in TASH NICU. **g** Photos from patients observed in this study wearing our device system.

Figure 5 compiles a collection of signals for thorough system validation with a clinical-grade reference device. Figure 5a plots a 10-second segment of filtered ECG data from a neonate in the study. The ECG waveform shows high signal quality, with easily differentiable P wave, QRS complex, and normal T-wave amplitude. Figure 5b compares the derived heart rate values between our device and a known reference (BioRadio) on a healthy adult, showing the accuracy of real-time heart rate determination. Real-time heart and respiration rates are derived from the raw ECG signal using the Pan-Tompkins algorithm for QRS-complex detection^14^. The ECG was sampled at 250 Hz, ensuring approximately 68 samples per heartbeat at the highest expected neonatal heart rate (~220 bpm), exceeding the recommended guidelines for robust QRS complex characterization and detection. Measured at 250 Hz, the raw ECG signal undergoes a 4th-order bandpass filter of 0.05–124 Hz and a 60 Hz noise rejection notch filter to isolate the desired frequency band. A 5-point derivative filter with a gain of 0.125 and processing delay of two samples and a consequent squaring of the filtered signal accentuates the QRS fiducial. The number of QRS peaks found in a 30 s moving window determines the heart rate. From the found peaks, a simple interpolation can identify the respiratory artifacts, where the resulting respiration rate is the rate of found maxima of the spline in the same moving window (Fig. S9). Representative denoised ECG waveforms comparing PCB electrodes and conventional gel electrodes, clearly showing superior signal amplitude and clarity with PCB electrodes, are provided (Fig. S10). SpO2 is derived from the measured absorbance of the red (660 nm) and infrared (940 nm) light emitted from the PPG sensor (Fig. S11). Similarly, the PPG was sampled at 50 Hz, providing ~13–14 samples per heartbeat, sufficiently meeting established Nyquist criteria and international standards for pulse oximetry, ensuring reliable monitoring of neonatal heart rates exceeding 200 bpm. Figure 5c shows satisfactory PPG signal quality with differentiable cardiac beats with distinguishable dichroitic notches from the REF and infrared (IR) waveform. Physiologically, the two wavelengths have different absorbance behavior from oxygenated hemoglobin and deoxygenated hemoglobin, with IR often having greater robustness. Sampled at 50 Hz, the recorded signals from the receptive photodiodes are subjected to a 0.5–9 Hz bandpass filter. PPG involves measuring the absorbance of red and infrared light during the cardiac cycle. Recording the light absorbed from each light source’s receptive photodetector, stable non-pulsatile (DC) and pulsatile (AC) components are divided. The ratio between each wavelength’s AC/DC components forms an absorbance ratio, R. The resulting ratio is paired with an experimentally derived calibration curve to estimate SpO2^15–18^. Figure 5d shows the results of the breath hold test on a healthy adult, demonstrating measurement agreement with a reference device (BioRadio). Figure 5e shows a segment of the temperature reading, which reaches stable values after 10 min of wear, as the encapsulated device at room temperature must warm to skin temperature. The real-time metric determination methods are integrated directly within the system alongside an embedded red flag detection algorithm. The app implements automated red flag detection using clinically validated thresholds for vital signs. Continuous monitoring enables early detection of abnormal trends in heart rate (<100 or >160 bpm), respiratory rate (<30 or >60 breaths/min), temperature (<36.5 °C or >37.5 °C), and blood oxygen saturation (<90%). The interface prioritizes critical alerts through a notification system that categorizes deviations based on severity and persistence. Real-time data visualization allows healthcare workers to track vital sign trends and assess intervention effectiveness, with data logging capabilities for post-hoc analysis of patient outcomes^19^. A simplified single-screen dashboard displays vital parameters with clear color-coding to indicate normal and concerning ranges, enabling quick patient assessment through both visual and vibration notifications to ensure critical information delivery in an unpredictable environment. An example of the real-time operation of the system can be viewed in Supplementary Video S1. Figure 5f exhibits a neonate undergoing ECG-based continuous monitoring, covered by a nest of wired electrodes connected to suction-based probes, limiting motion and causing visual distress to parents. Figure 5g compiles photos from patients observed in this study, demonstrating comfortable, appealing, and accurate monitoring, improving upon traditional methods. The attending physician saw no adverse conditions resulting from the application of the device.

To assess the usability and acceptability by Ethiopian clinicians and caretakers, a one-page survey was completed by parents of neonates before they were discharged from the hospital. Alongside this, parents were interviewed, and their feedback was recorded by nurses on-site. The feedback, originally provided in Amharic, was translated into English. The summarized questions and survey results are presented in Table 2. The survey focused on comparing current measurement techniques with the biopatch developed in this work. Results revealed that 48% of parents found the biopatch easy to use, while 84% expressed a willingness to use it at home, preferring it over manual measurements. Additionally, 89% stated that the biopatch was user-friendly. Despite these positive findings, parents highlighted the need for technical support, with 89% agreeing that expert training is essential for effective use. Furthermore, 58% of parents indicated they would prefer the biopatch for future use. While the biopatch received positive feedback on usability and acceptability, concerns were raised regarding the need for training and the potential challenges associated with using electronic devices in a neonatal care setting.

**Table 2:** Summary of biopatch survey results and patient feedback on usability, acceptability, and technical requirements

Question Summary	Survey Results	User Inputs Summary
Difficulty in using manual measurements	52% found it difficult or very difficult; 48% found it easy	Parents found manual measurements time-consuming and disruptive to their baby’s rest
Using manual measurement tools at home?	63% said they would use them at home	Parents preferred continuous monitoring tools to avoid frequent disturbances caused by manual checks
Difficulty in using the biopatch?	0% reported “very difficult”; 11% found it difficult; the rest found it easy	Parents noted that the device requires time and expert training to use independently
Using the biopatch at home?	84% said they would use it at home	Parents were satisfied with the continuous monitoring and reduced need to wake the neonates
Confidence in monitoring technique	53% were confident in using manual measurement techniques	Parents expressed concerns about the accuracy of the biopatch as compared to manual methods
Preference for future use	58% preferred the biopatch over manual measurements for future use	Concerns were raised about device aesthetics and fears of electronic components affecting neonates’ health
Need for technical support for biopatch	89% agreed technical support is needed	Parents emphasized the need for expert training and support to use the device effectively
Perception of system, ease of use	74% agreed the biopatch is quick to learn and use	While the device was seen as user-friendly, concerns were raised about durability and sustainability

## Discussion

While continuous monitoring stands as the standard in high-resource healthcare, current solutions are expensive, energy-consumptive, and require rigorous training and infrastructure for operation. Affordable, continuous physiological monitoring systems are a crucial tool for early deterioration detection and intervention of at-risk neonates, but few studies have targeted this challenge to improve the quality, access, and efficiency of care to reduce neonatal mortality in low-resource settings^20,21^. Previous wearables have been designed to condense the complicated setups in NICU monitoring into miniaturized wearables for facilitated monitoring^4–6^; however, they encounter problems with device longevity with either single-use or short-term use before replacement. Systems that are specifically designed for remote and underserved settings need further development and attention. In this work, we discussed the development of a wearable system tailored for use in Ethiopian hospitals with design considerations on nurse availability, internet, resources, and parental skepticism towards digital devices. Currently, nurses at the Tikur Anbessa NICU share monitoring tools between several patients for periodic manual measurement of vital signs. These rounds disturb and distress the observed patients, causing the inaccurate reporting of resting heart and respiration rate, as seen from the results of our comparison between our system’s reported values and both manual and ECG-derived values. Our system, though it also uses an elastomer-based, reusable adhesive to attach to the skin, has a lifecycle plan with replaceable electrodes. Our system can withstand a month’s worth of removal and replacements for a single patient, where once the adhesive is no longer suitable for use, it can simply be replaced with a new, low-cost adhesive electrode patch. In addition, the current monitoring protocol for blood oxygenation uses a clip-style pulse oximeter designed for adults that is insufficient for continuous monitoring due to its strong application pressure and incompatibility with infant physiology. As a result, we explored the option for continuous blood oxygen monitoring from the forehead, which is favorable for its stable location and reliable vascular density. In vivo testing results demonstrated high levels of agreement in real-time vitals determination, with reliable red flag detection to assist in identifying deterioration. Overall, parents were impressed by and appreciated the ease of use of the system and its continuous functionality without disturbance to the infant. Although great strides and considerations were made in this study, there are limitations to address for future studies. First, due to the nature of novel flexible technology, in future iterations, we would suggest the use of a hybrid device combining a rigid operating system with flexible PCB electrodes to ruggedize the main reusable portion while maintaining the use of the low-cost, high-sensitivity electrodes. In addition, several parents were wary of the appearance of the device; therefore, improved packaging of the devices with reduced exposure of electrical components will improve acceptability. Finally, the biggest concern shared by parents was the need for training to operate the system correctly. Developing a simple yet comprehensive training plan should help alleviate concerns. As a pilot study, this experiment observed the practical application of our system in 25 patients.

In summary, this paper reports on the development of a soft, all-in-one, wearable, and user-friendly system to allow high-quality, continuous monitoring of neonates in Ethiopia. In vivo testing results show high levels of agreement in real-time vitals determination, with reliable red flag detection to assist in identifying deterioration. The system’s ability to provide continuous monitoring, reducing disruptions to neonates, represents a significant improvement over traditional manual measurement methods. A set of human subject studies captures the main advantages of our device, minimizing stress and disturbances to babies, eliminating the need to wake them up for routine checks. Overall, the wearable system is a superior alternative to manual measurements and is the preferred method of measurement for neonatal care. Based on findings from this pilot, an additional 100 neonates will be recruited in future studies to evaluate the second generation of the device. The results of this study will serve as a foundation for calculating sample sizes for larger-scale research on this technology: soft membrane devices for neonatal health monitoring.

## Methods

### Device fabrication

The circuit schematics and board designs for both the chest and forehead devices were designed using Altium, considering optimal signal integrity through multi-layer printed circuit board (PCB) architecture and noise isolation techniques, and fabricated using an ISO 9001-compliant 3rd party manufacturer. The surface mount microcontroller, sensors, passive, and antenna components were off-the-shelf components and assembled on the board using reflow soldering techniques (see the details in Fig. S12). Firmware was developed using Segger Embedded Studio and flashed onto the devices to test functionality. To protect the electronics, they are encapsulated in an elastomeric coating (Ecoflex 0030, Smooth-On). For the chest device, the assembled PCB was placed atop a glass slide prepared with a thin layer of cured elastomer and uncured elastomer was distributed on top of the board and cured in a 60 °C oven for 40 min to create a water-tight seal. A second layer of elastomer was applied atop the device to ensure complete encapsulation. The elastomer-coated slide was prepared with a thin coating of a sacrificial layer (Ease Release, Smooth-On) and 5 g of elastomer spin coated at 300 revolutions per minute (RPM) for 15 s and baked for 40 min in a 60 °C oven. After complete curing, the device was carefully removed from the glass slide and trimmed to remove excess elastomer. Next, the encapsulated device was adhered to a prepared bandage coated in a biocompatible and reusable elastomeric adhesive using epoxy (JB Weld Quickset Epoxy). The bandage was fabricated from a medical-grade fabric substrate (3 M 9907 T) and 10 g of high-tack elastomer (Elken Silbione) was spin coated onto the adhesive-side of the tape at 700 RPM for 1 min. The tape was then set to cure for 45 min in a 60 °C oven. Adhered to the underside of the fabric substrate was a custom serpentine PCB electrode with a stiffened fastener that can be inserted into the flat flex cable (FFC) connector of the main PCB. For the forehead device, the assembled device was first laid atop and adhered to its battery using epoxy (JB Weld Quickset Epoxy). Next, black-colored elastomer was deposited atop the entirety of the front of the device, carefully avoiding the surface of the photoplethysmogram (PPG) sensor and set to cure for 40 min in a 60 °C oven. After an initial layer, a second layer is similarly distributed and cured. Finally, the bottom side of the exposed PCB was coated in a layer of elastomer and small piece of hook-sided Velcro was adhered to the back side of the battery with super glue (Gorilla Super Glue Gel). To prepare the mounting headband, an equally sized piece of loop-sided Velcro was adhered to the center of the front of a wide banded neonatal headband. To resist headband movement, a small knot was sewn above the Velcro to keep all layers of the headband stable.

### Integrated electronics and communications

A simplified block diagram of the electronic circuitry, bill of materials, and component layout for both the chest and forehead devices are detailed in Figs. S13–S14. To maintain consistency, both devices operate using the same microcontroller (Nordic Semiconductors, nRF52832), chosen for its ultra-low-power Bluetooth Low Energy (BLE) capability and ARM Cortex-M4 architecture for optimal processing and integration with biomedical sensors. Electrocardiogram (ECG) sensing was measured by a 24-bit biopotential analog-to-digital converter (ADC) (Texas Instruments, ADS1291) sampled at 250 Hz and interfaced to the microcontroller using SPI protocol. Skin temperature sensing was measured using a 12-bit medical grade temperature sensor (Texas Instruments TMP117) and sampled at 1 Hz. The temperature sensor was interfaced to the microcontroller using I2C protocol. For the forehead device, a two-channel PPG sensor (MAXIM, MAX30102) was interfaced with I2C protocol with a 50 Hz sampling rate. To address potential signal interference, careful spatial separation and dedicated shielding were employed, confirming minimal cross-interference between electrode sets. Furthermore, theoretical analysis and experimental validation of Bluetooth Low Energy (BLE) transmission indicated negligible collision probability and minimal data loss, supported by a low transmission power (−40 dBm), short packet duration (3.73 ms), small payload size (5 bytes), and extended advertising intervals (150 ms). Explicit quantitative BLE performance metrics—including RSSI typically between −50 and −60 dBm, packet loss rates below 0.1%, and synchronization latency under 10 ms—are detailed in Figure S15, validating reliable wireless operation.

### Remote data saving

Due to limited internet penetration in Ethiopia, internet connectivity is not required for real-time metric calculations. The system employs an offline-first architecture, where real-time vitals are computed locally using edge processing techniques, ensuring uninterrupted functionality in low-connectivity environments. Data is stored in an encrypted SQLite database on the tablet, and an asynchronous synchronization mechanism uploads the data to a cloud server when connectivity is restored, preserving data integrity and continuity.

### Time synchronization

When using multiple wireless devices, it is important to obtain accurate data time stamping and synchronization to ensure detailed and correct recordings. When a single tablet collects from several devices simultaneously, the risk for data dropout and lagging can compound to increase inter-device timing deviations. In addition, although individual smart devices have their own internal clocks that are fairly accurate to true time, they can deviate up to several seconds from the ground truth. Therefore, our system leverages the existing internet connection to synchronize with cloud servers’ atomic clocks using Network Time Protocol (NTP) at app startup and at intermittent intervals, enabling high-fidelity biomedical signal acquisition and minimizing timing deviations to under 10 ms for precise multi-device alignment and correlation. Shown in Fig. S16, the iterative time correction allows consistent and reliable timestamping. When internet connectivity is limited, time synchronization is paused and recalibrated once reconnected. Due to the real time monitoring use case of this device system, highly accurate time synchronization is not mandatory.

### Device sanitation and electrode replacement

The reusable device can be sanitized by spraying isopropyl alcohol onto the silicone adhesive and allowing it to dry. While the adhesive has strong adherence after many application and sanitation cycles, if the adhesive of the patch is no longer sticky, the electrodes can no longer collect good signals, or the processing unit needs to be used by a different patient, the electrode PCB can be removed from the FFC connector and attached to a new electrode patch with epoxy.

### Adhesive strength testing

Figure S17 shows the experimental setup to measure the strength of our elastomer-based adhesive. To conduct the 180° peel test, the skin on the forearm was prepared by shaving the applied area mimic smooth infant skin and sanitized with an alcohol wipe. A 3.8 cm by 10.2 cm piece of prepared tape was attached to the skin, with 2.54 cm of length folded to create a 1.27 cm pull tab to be connected to the force gauge, facing towards the bottom of the observed length. After being placed on the skin, it was rubbed gently to ensure conformality to the skin and left to settle into the skin for 5 min. Next, the tab was clamped to the force gauge (Mark-10) and peeled off the skin at 180° at a rate of 2 cm/s. After removal, the tape was sanitized using a fine mist of isopropyl alcohol and left to dry for 30 s. The experimental test studied *n* = 20 observations.

### Software-only motion handling

To address motion artifacts without modifying additional hardware, we derived a motion-quality index (MQI) from the recorded ECG where the signal was band-pass filtered (0.05–124 Hz, 250 Hz sampling). The summarized datasets appear in Fig. S18. MQI was computed as the normalized average of the Hilbert envelope of baseline wander (<0.5 Hz) and short-time energy in 2 s windows advanced every 0.5 s. A data-driven threshold (median + 3×MAD) was applied to mark high-motion epochs. Additionally, to further quantify internal consistency, three metrics were computed before/after MQI-gating, when PPG was available: 1) absolute difference between PPG pulse rate and ECG heart rate (|PR-HR | ), 2) cardiac-band SNR of PPG (0.8-3 Hz power / 3-10 Hz power), and 3) ECG-PPG wavelet coherence in the 0.8–3 Hz band.

### Human subject study

This study was approved by the Ethiopian Food and Drug Administration under reference No: 02/25/33/42 on April 3, 2023; with Institutional Review Board reference No: 021/21/Pedi. Parents of neonates admitted to the Tikur Anbessa (Black Lion) Specialized Hospital (TASH) Neonatal Intensive Care Unit in Addis Ababa, Ethiopia, who met the inclusion criteria were approached and invited to participate. The inclusion criteria encompassed both term and preterm neonates aged 0–7 days admitted to the NICU at TASH. Exclusion criteria included critically ill neonates, surgical patients with attached devices near wound sites, and cases where the attending physician recommended against inclusion. Using Schmettow’s methodology for sample size estimation^22^, a total of 25 neonates (*n* = 798 data entries) were recruited to assess the first generation of the device. The key characteristics of the recruited neonates are summarized in Table 3. Upon receiving written consent, trained nurses placed the device system on the neonate’s chest to record heart rate, respiration rate, SpO2, and temperature using the device’s smartphone app. Written consent was obtained to publish the details, images, and videos of this study. A biomedical engineering research scientist was on-site to assist the nurses throughout the process. Simultaneously, nurses performed manual measurements as part of standard care protocols. Temperature was measured using a thermometer, heart rate and oxygen saturation were measured with auscultation and a pulse oximeter, and respiratory rate was measured by manually counting for 60 s. Over the study period, any complications with the device system, such as loss of communication, inaccurate measurements, loss of adhesion, or irritation around the adhesion site, were recorded using a device information questionnaire. If any irritation was observed by parents or nurses, the device was gently removed. The device was removed if the neonate was discharged from the NICU at any given point during the study. A total of 592 h of data were collected at TASH NICU. At the end of the study, parents completed a qualitative survey to evaluate the acceptability and usability of the device.

**Table 3:** Biopatch usability and feasibility study—participant summary

Characteristic	Summary
Age (days)	Median: 3.0 Range: (1,6)
Participation length (hours)	Median: 31.0 Range: (5, 84)
Reason for admission	‘Early onset neonatal sepsis’: 12 ‘Hyperbilirubinemia’: 3 ‘Meconium aspiration syndrome’: 3 ‘Neonatal jaundice + EONS + Hyperkalemia’: 1 ‘Congenital conditions (Down Syndrome, CHD, ARM)’: 3 ‘Birth complications (PNA, HIE, Chorioamnionitis)’: 3
Reason for leaving	‘Discharged from the unit’: 21 ‘The grandmother monitoring the neonate refused’: 1 ‘Problem with device’: 1 ‘Withdrawn from the study’: 2
Condition of neonates	‘Stable’: 21 ‘Sub-critical’: 4

## Supplementary information

2. Rev2_SI

Supplementary Video 1

## Data Availability

The data that support the findings of this study are available in the supplementary material of this article.
