# Supplementary material for: Soft, all-in-one, nanomembrane wearable system for advancing neonatal health monitoring in Ethiopia
Source: NPJ Digit Med. 2025 Sep 25;8:575. doi: 10.1038/s41746-025-01974-8 (PMC12462490; doi:10.1038/s41746-025-01974-8)

Supplementary Data

Soft, All-in-One, Nanomembrane Wearable System for Advancing Neonatal Health Monitoring in Ethiopia

Lauren Zhou[1,2,†], Michele Joseph[3,4,†], Yoon Jae Lee[2,5,6], Diva Yadav[1,2], Likhit Nayak[1], Julia Woodall[1], Jared Matthews[1,2], Ira Soltis[1,2], Firehiwot Markos Mekuria[7,8], Kullehe Haddis Yeshanew[7,8], Yonas Kebede Mamo[4,7], Abebaw Fekadu[4,7], Asrat Demissie[4,7,8], Rudolph Gleason[1,9,*], and Woon-Hong Yeo[1,2,9,10,*]

[1]George W. Woodruff School of Mechanical Engineering, College of Engineering, Georgia Institute of Technology, Atlanta, Georgia, USA
[2]Wearable Intelligent Systems and Healthcare Center (WISH Center) at the Institute for Matter and Systems, Georgia Institute of Technology, Atlanta, GA, 30332, USA
[3]Center of Biomedical Engineering, Addis Ababa Institute of Technology, Addis Ababa University, Addis Ababa, Ethiopia
[4]Center for Innovative Drug Development and Therapeutic Trials for Africa College of Health Sciences, Addis Ababa University, Addis Ababa, Ethiopia
[5]School of Electrical and Computer Engineering, Georgia Institute of Technology, Atlanta, GA, 30332, USA
[6]Department of Computer Science, Georgia State University, Atlanta, GA, 30303, USA
[7]Tikur Anbessa Specialized Hospital, Addis Ababa University, Addis Ababa, Ethiopia
[8]Neonatal Intensive Care Unit (NICU), Department of Pediatrics and Child Health, College of Health Sciences, Addis Ababa University, Addis Ababa, Ethiopia
[9]Wallace H. Coulter Department of Biomedical Engineering, College of Engineering, Georgia Tech and Emory University School of Medicine, Atlanta, Georgia, USA
[10]Korea KIAT-Georgia Tech Semiconductor Electronics Center (K-GTSEC) at the Institute for Matter and Systems, Georgia Institute of Technology, Atlanta, GA, 30332, USA
[*]Corresponding author - E-mail: whyeo@gatech.edu or rudy.gleason@me.gatech.edu
[†]The authors contributed equally to this work

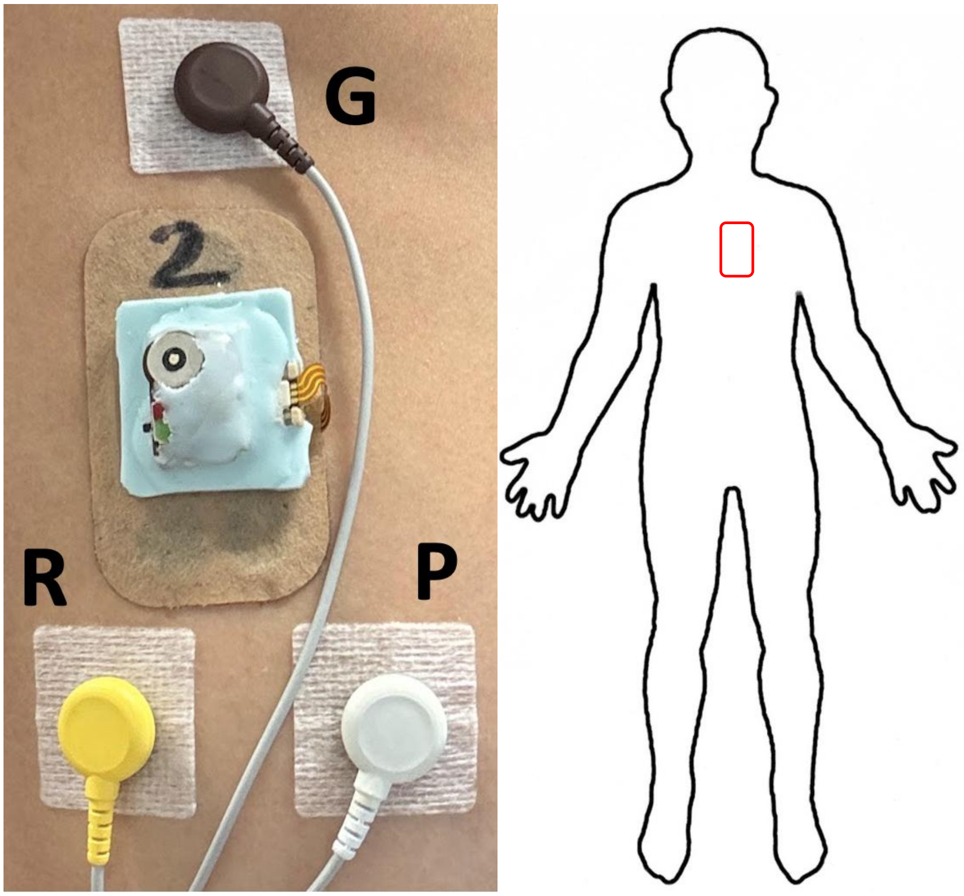

Figure S1. An experimental setup compares the proposed PCB electrodes with reference gel electrodes on unprepped skin slightly left of the sternum above the heart. The electrode placement utilized a modified V2 setup, with the ground (G) electrode above the heart, the reference (R) electrode below and to the right of the heart, and the positive (P) electrode below and to the left of the heart. The study was conducted on a healthy female adult.

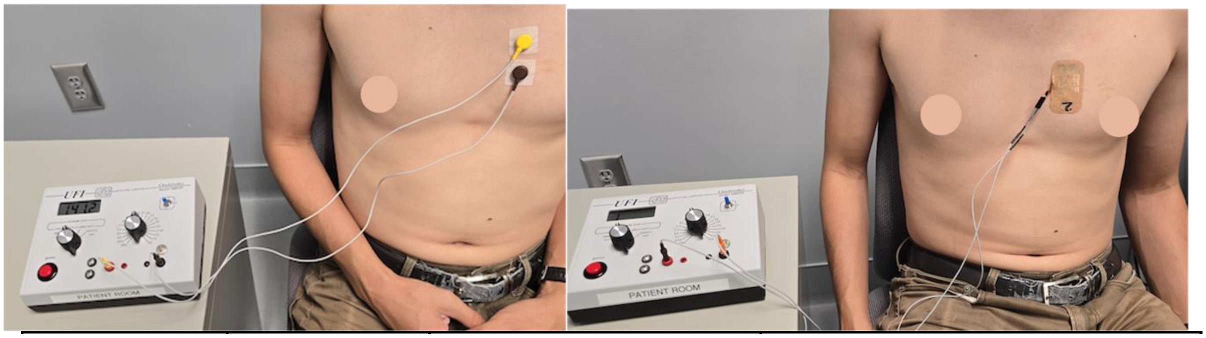

	Impedance (kOhm)	Unit Area (mm^2)	Impedance Density (kOhm / cm^2).
Gel electrode	0.145	225	0.064
Dry gold electrode	1	81	1.2

Figure S2. Photos of the impedance measurement setup with conventional gel electrodes (left) and dry gold electrodes (right).

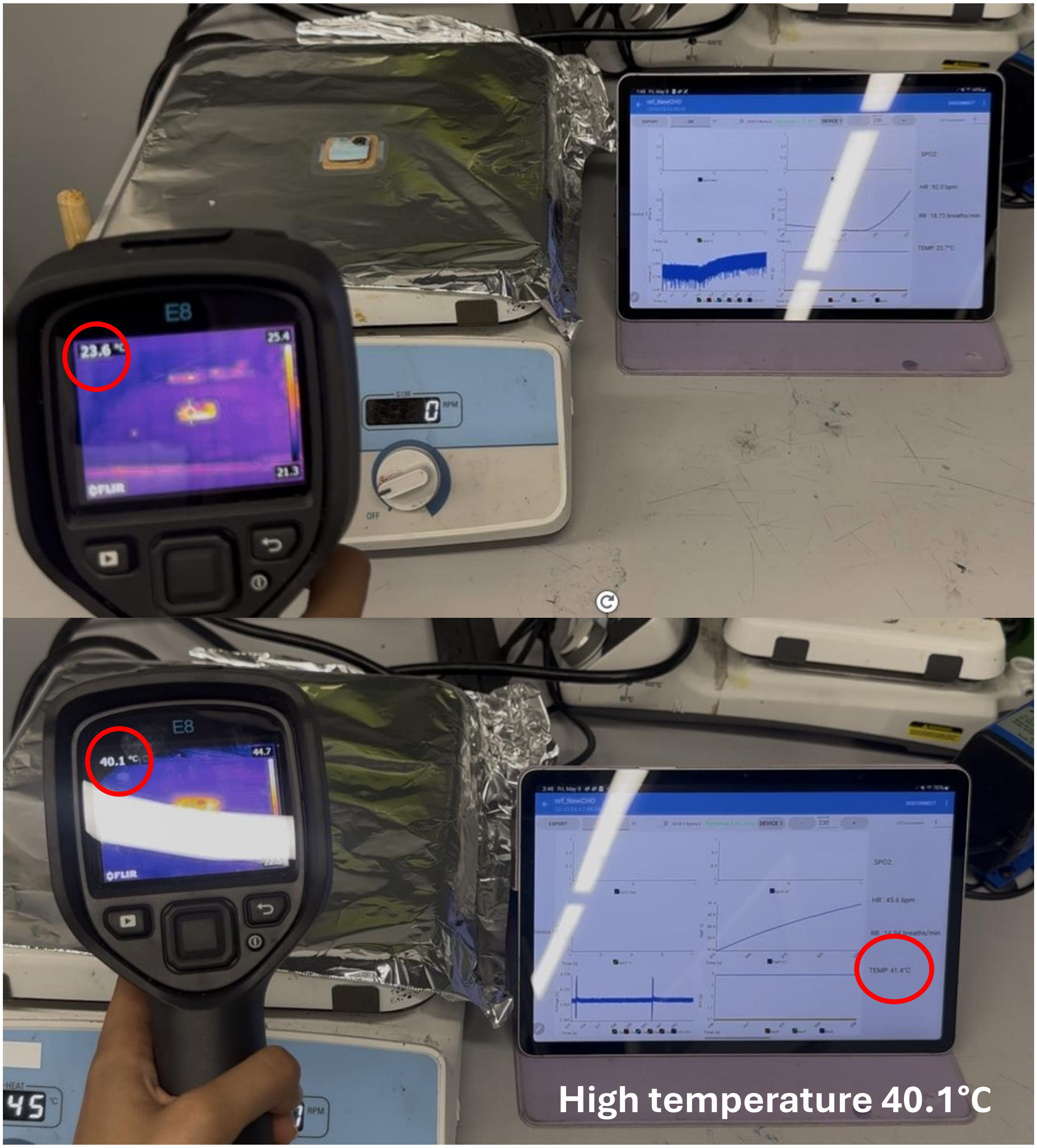

Figure S3. Experimental setup for the temperature sensor tracking performance. The device was placed on a hot plate with varying temperatures from 23°C to 43°C. Recorded with a video, a FLIR E60 infrared thermal imaging camera was pointed towards the hot plate surface alongside the running tablet. Afterwards, going through the recorded video, the temperature pulled from the infrared camera and the reading from the tablet were recorded and plotted.

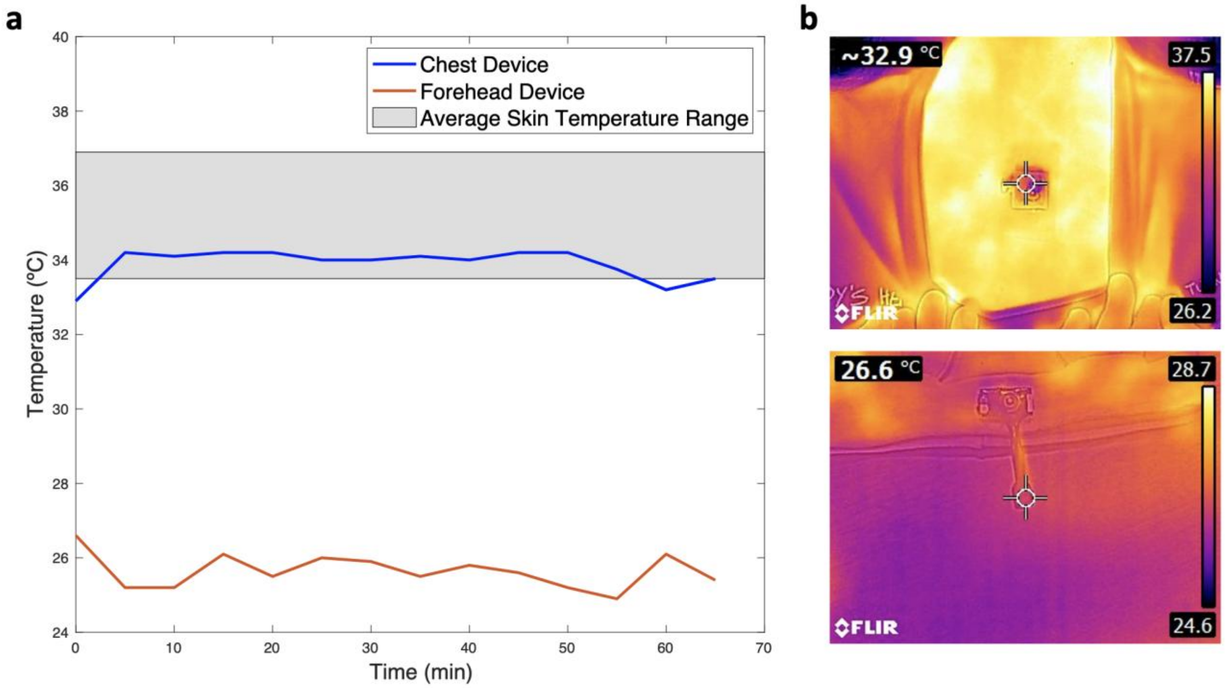

Figure S4. Results and experimental setup to observe the thermal behavior of the devices, showing negligible heat generation from the device. The two devices were worn by a healthy adult. The chest device was worn continuously, and the forehead device was removed every 5 minutes. (a) The chest device does not surpass the average skin temperature, and the forehead device retains the same heat as the cloth headband. (b) The temperatures logged were recorded by a FLIR E60 infrared thermal imaging camera.

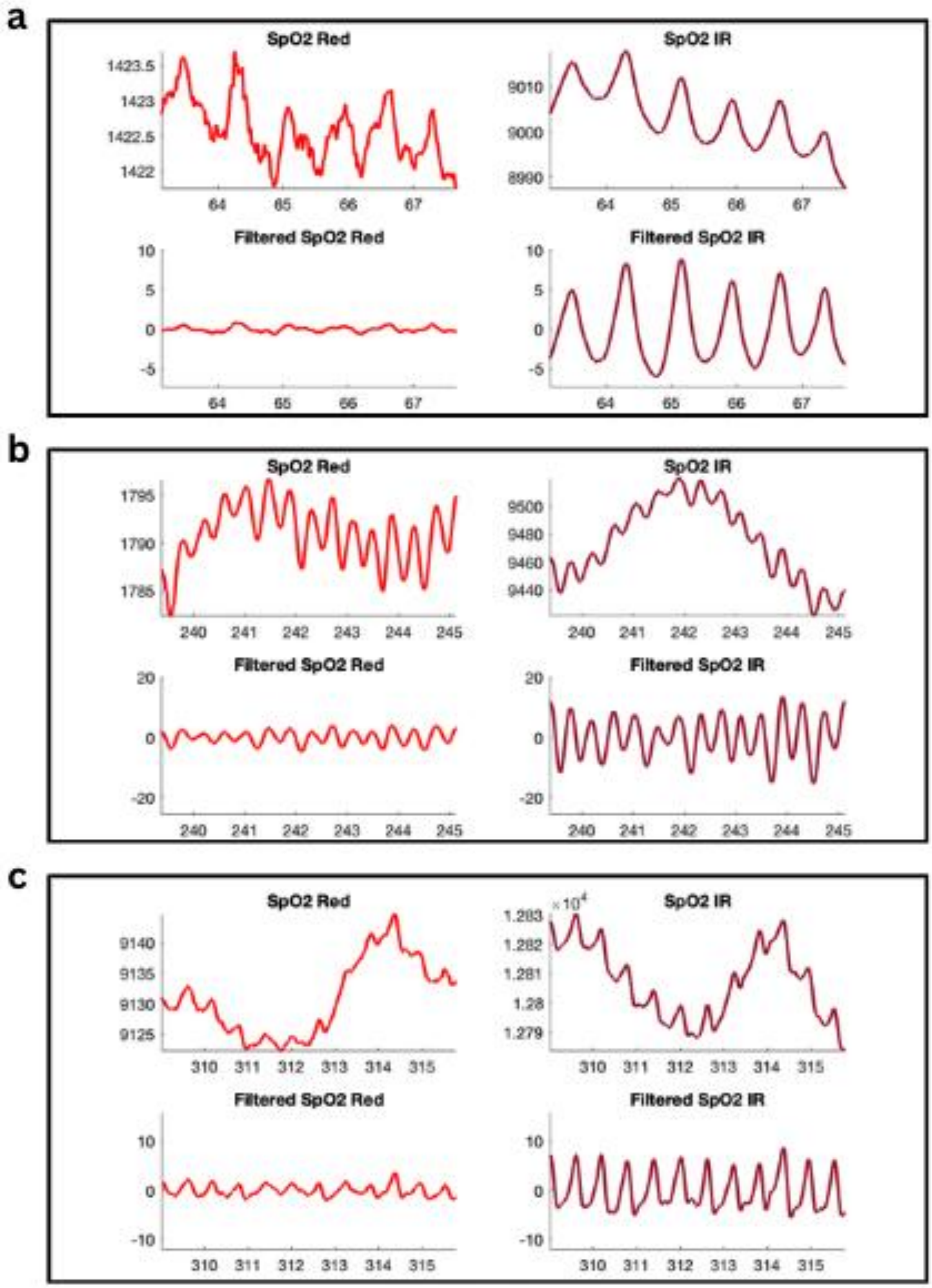

Figure S5. A stable, high-fidelity PPG signal was measured using a forehead-mounted device on a healthy adult while on a patient who was (a) standing still, (b) walking, and (c) running.

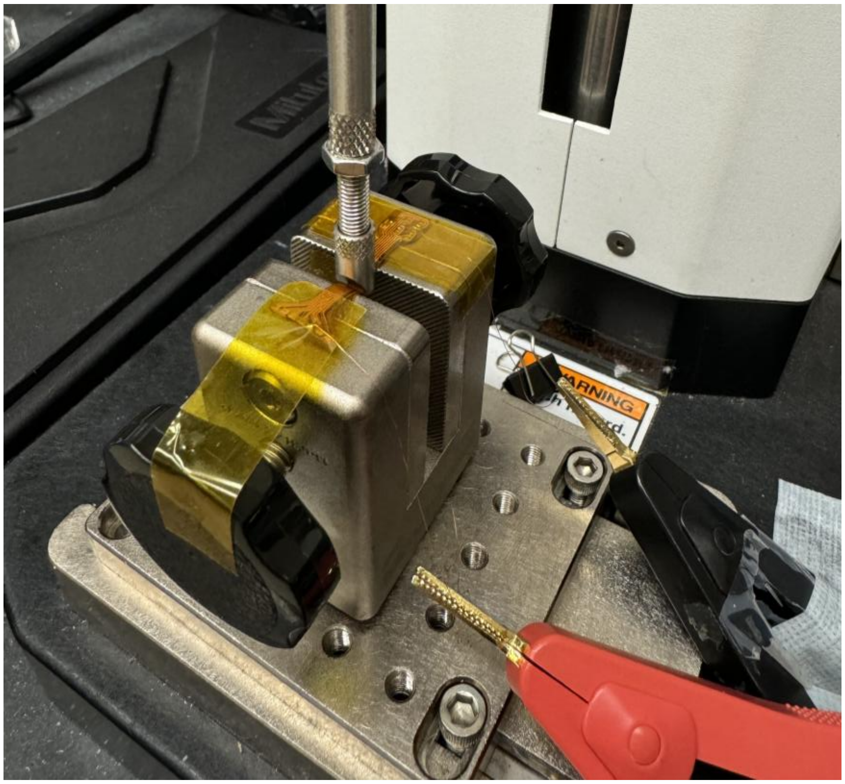

Figure S6. Forehead printed circuit board sensor extension 180° bend test experimental setup. The device was allowed to stay straight across the lower supports, where a plunger was pushed into the substrate 4 cm to allow a complete 180° longitudinal bend. The bend cycle duration was 4 seconds.

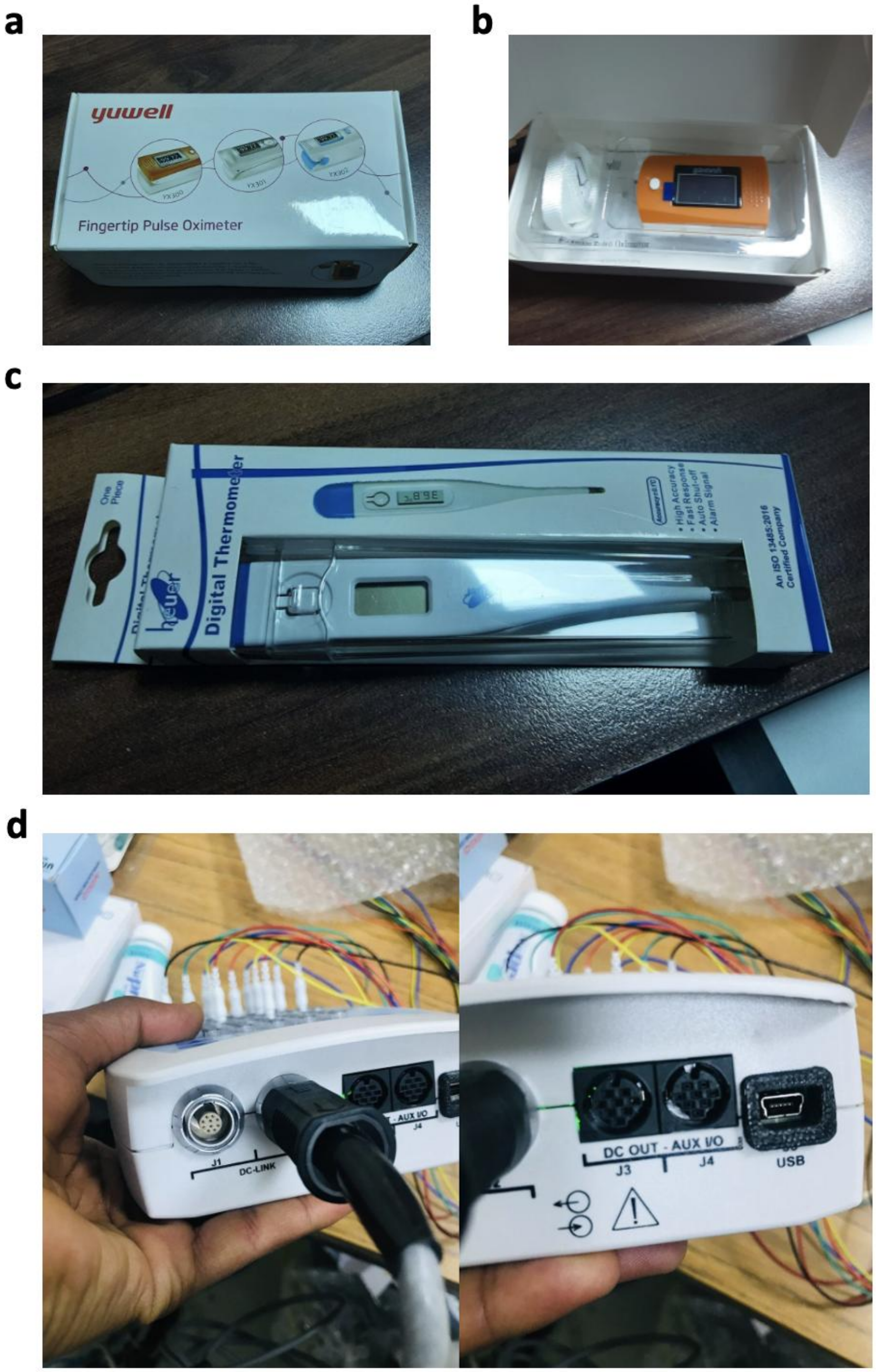

Figure S7. (a)-(b) Reference pulse oximeter for manual heart rate and SpO2 measurement. (c) Reference axillary thermometer for temperature sensor comparison. (d) Reference ECG patient monitor (unknown brand)

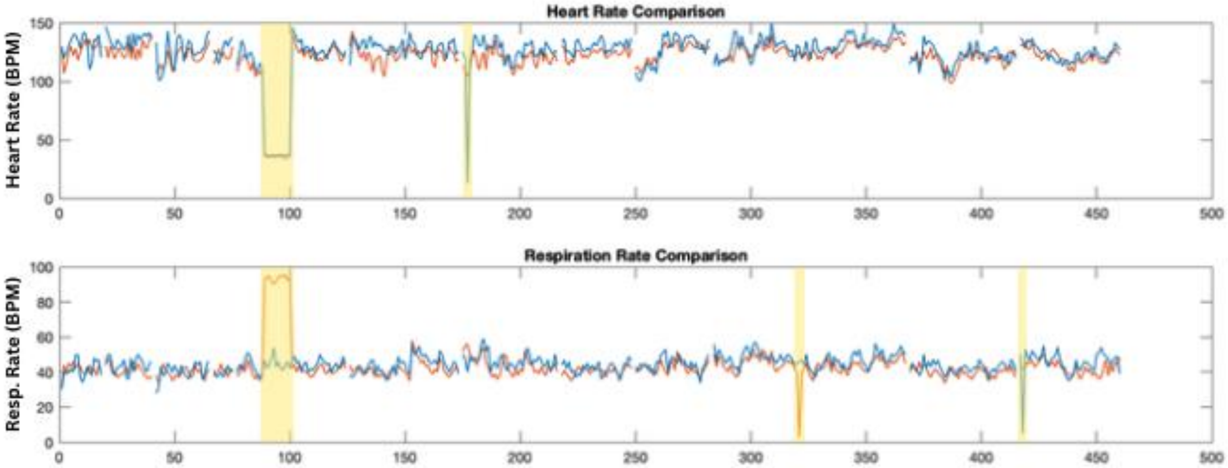

Figure S8. Complete logged data. Data entries that were out of normal range for neonates (HR<100 BPM, RR<34 BPM | RR>60 BPM, SpO2>100%) were removed. Such removed values are indicated in the highlighted regions.

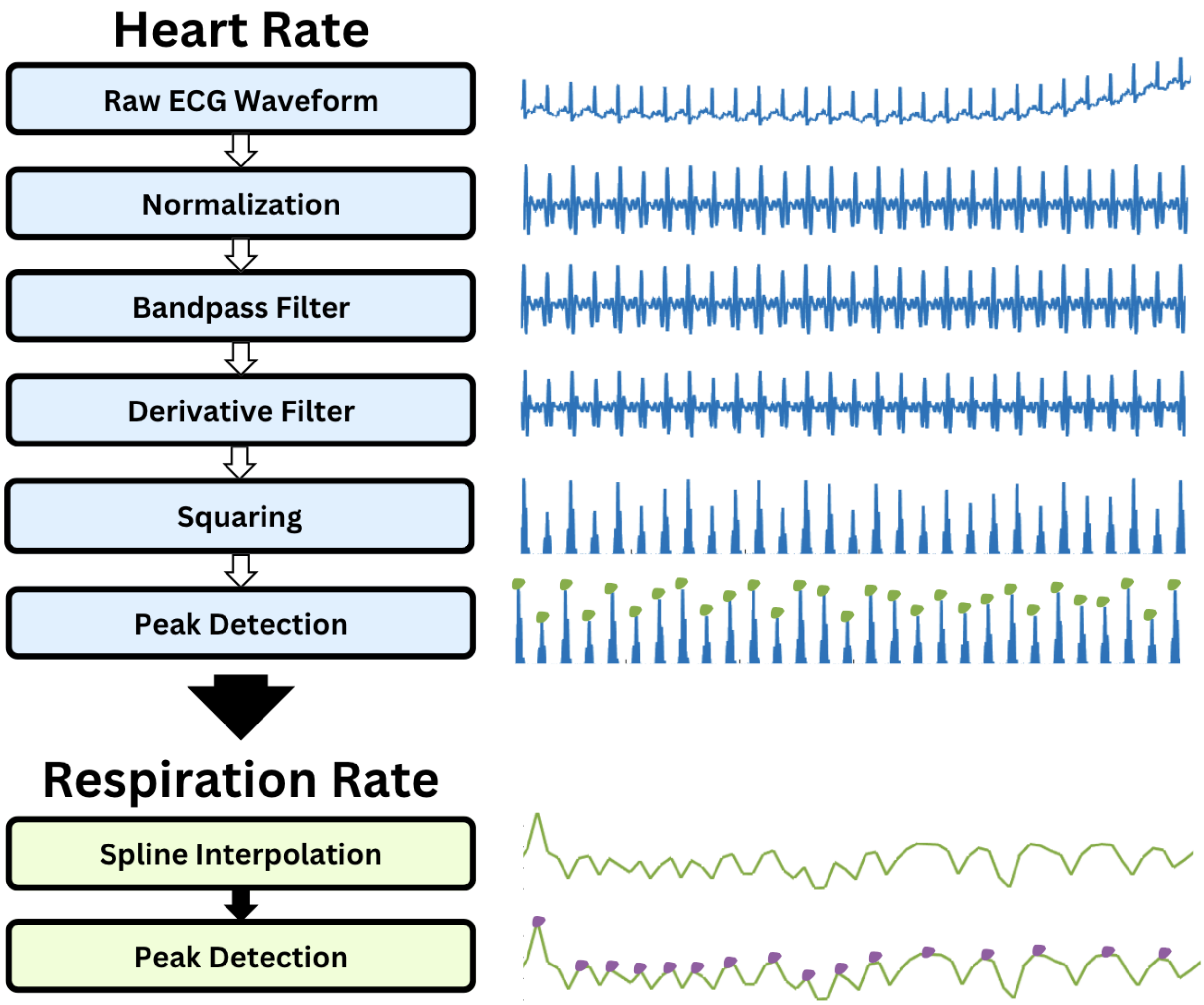

Figure S9. Pan-Thompkins algorithm for real-time heart rate and respiration rate determination from ECG waveforms.

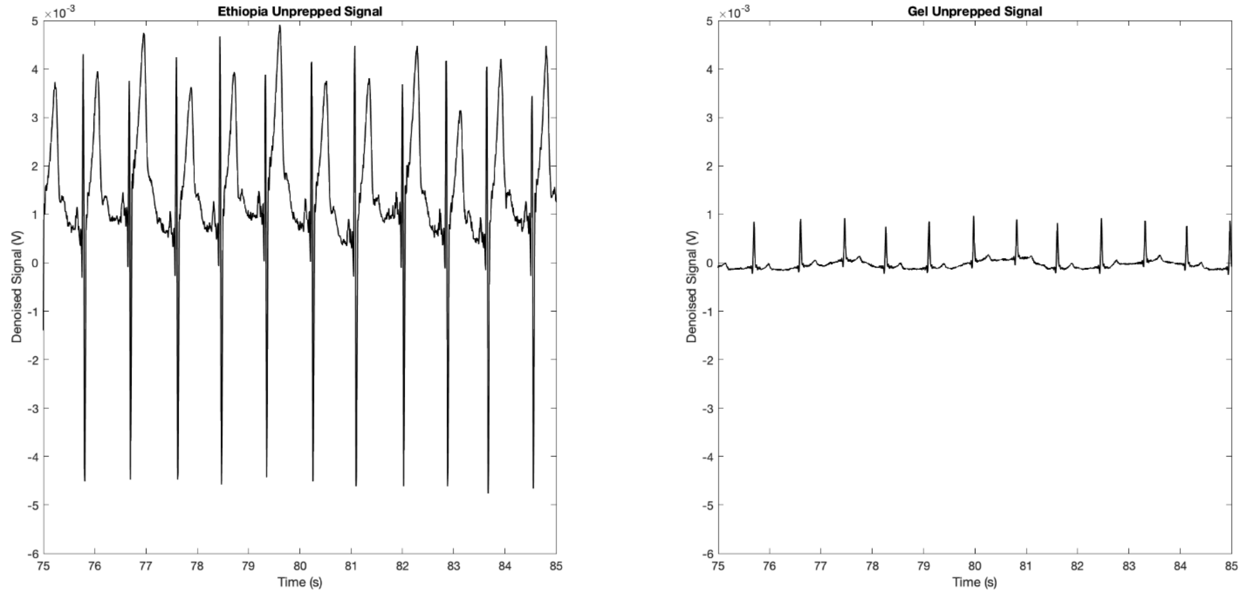

Figure S10. 125 Hz ECG waveforms comparing the developed PCB electrodes ("Ethiopia Unprepped Signal", left) and conventional Ag/AgCl gel electrodes ("Gel Unprepped Signal", right).

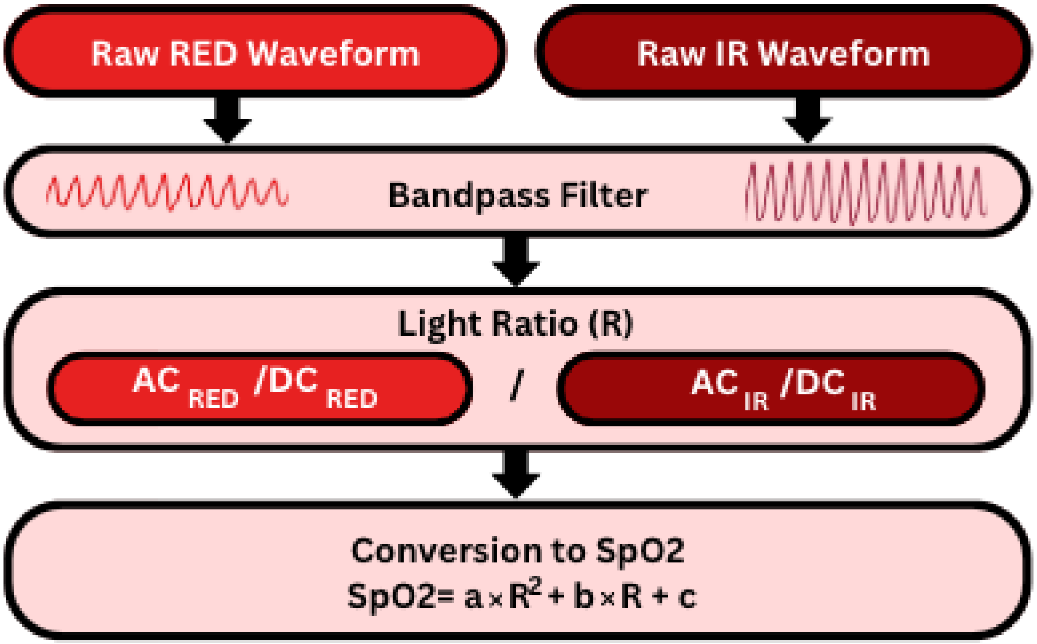

Figure S11. PPG processing method for real-time peripheral capillary SpO2 determination.

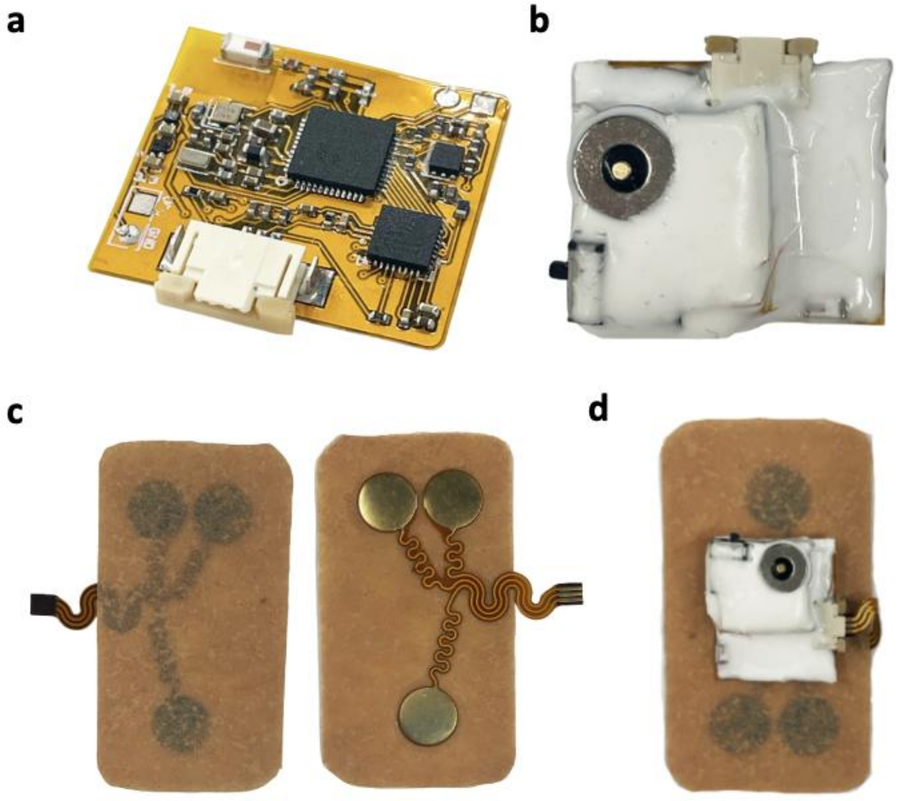

Figure S12. (a) The devices are assembled with the microcontroller unit, sensors, and passive electrical components soldered onto the custom-printed circuit boards. (b) After assembly, the elements are encapsulated with a soft silicone rubber (Smooth-On Ecoflex 00-30) to protect the electrical components from water and dust. (c) The electrodes are attached to a fabric band-aid-like patch that has a naturally adhesive biocompatible silicone-based elastomer on the adhesive side. The properties of the adhesive elastomer allow it to be sanitized, reusable, and gentle on the skin. (d) The device is epoxied to the top of the fabric patch, and the electrodes are attached to the connector to finalize the fabrication of the device.

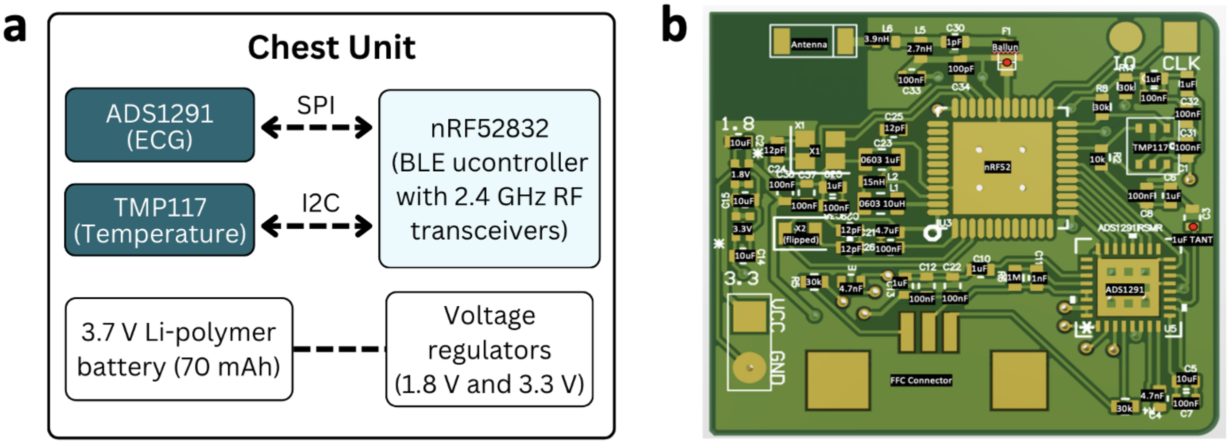

c

Description / Value	Purpose	*Qty	*Mfg Part #	*Unit Price
**ACTIVE**				
NCP177BMX330TCG	Voltage Regulator (3.3V)	1	NCP177BMX330TCG	$0.51
NCP177AMX180TCG	Voltage Regulator (1.8V)	1	NCP177AMX180TCG	$0.51
NRF528XX-QFAA	Microcontroller	1	NRF52832-QFAA-R	$5.48
ADS1291IRSMR	Bioimpedance Sensor	1	ADS1291IRSMR	$8.74
TMP117	Temperature Sensor	1	TMP117AIDRVR	$7.16
**PASSIVE**				
CHIP-ANTENNA (Chest)		1	2450AT18A100E	$0.79
2450FM07A0029	Ballun Filter	1	2450FM07A0029T	$0.28
1.0pF (0402)		1	CC0402CRNPO9BN1R0	$0.04
12pF (0402)		4	GRM1555C1H120GA01D	$0.03
100pF (0402)		1	C0402C101J5GACTU	$0.04
1nF (0402)		1	GRM1555C1H102JA01D	$0.04
4.7nF (0402)		2	GRM155R71H472JA01D	$0.04
100nF (0402)		12	C0402C104K8PACTU	$0.02
1uF (0402)		6	GRM155R61A105KE15D	$0.03
TANT 1uF (0402)		1	298D105X0010K2T	$1.97
1uF (0603)		1	GRM155R61A105KE15D	$0.03
4.7µF (0402)		1	C1005X5R1A475M050BC	$0.04
10uF		4	CL05A106MP5NUNC	$0.02
2.7nH (0402)		1	LQG15HS2N7S02D	$0.17
3.9nH (0402)		1	LQG15HN3N9S02D	$0.17
15nH (0402)		1	LQG15HS15NJ02D	$0.17
10uH (0603)		1	LBMF1608T100K	$0.29
10k (0402)		1	ERJ-2RKF1002X	$0.04
30k (0402)		4	ERJ-2RKF3002X	$0.02
1M (0402)		1	RC0402FR-071ML	$0.02
Crystal_GND24		1	ABM12W-24.0000MHZ-8-D1X-T3	$1.05
32.768 kHz		1	ABS07-32.768KHZ-T	$0.91
3-pin FFC Connector		1	WM10935TR-ND	$0.90
			total	$30.14

Figure S13. Chest device (a) detailed block diagram, (b) component layout, and (c) bill of materials. Active components are off-the-shelf components. Passive components are in the 0402 or 0603 sizing package. The total cost of the chest device without electrodes is $30.14.

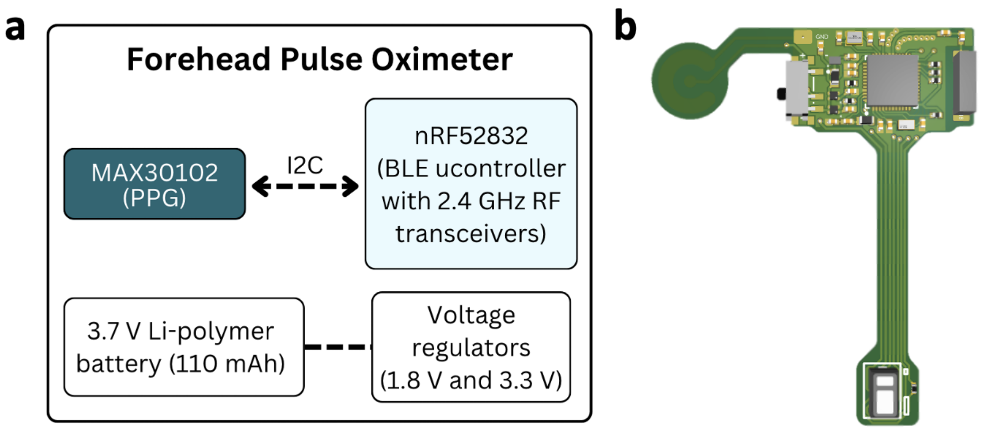

c

Description / Value	Purpose	*Qty	*Mfg Part #	*Unit Price
ACTIVE				
NCP177BMX330TCG	Voltage Regulator (3.3V)	1	NCP177BMX330TCG	$0.51
NCP177AMX180TCG	Voltage Regulator (1.8V)	1	NCP177AMX180TCG	$0.51
NRF528XX-QFAA	Microcontroller	1	NRF52832-QFAA-R	$5.48
MAX30102	RED PPG	1	MAX30102EFD+	$11.40
PASSIVE				
CHIP-ANTENNA (Forehead)		1	732-74889402450TR-ND	$1.24
1.0pF (0402)		2	CC0402CRNPO9BN1R0	$0.04
12pF (0402)		4	GRM1555C1H120GA01D	$0.03
100pF (0402)		1	C0402C101J5GACTU	$0.04
100nF (0402)		2	C0402C104K8PACTU	$0.02
1.0uF (0402)		3	GRM155R61A105KE15D	$0.03
4.7uF (0402)		1	C1005X5R1A475M050BC	$0.04
3.9nH (0402)		1	LQG15HN3N9S02D	$0.17
10uH (0603)		1	LBMF1608T100K	$0.29
15nH (0402)		1	LQG15HS15NJ02D	$0.17
0 Ohm (0402)		2	RMCF0402ZT0R00	$0.10
10k (0402)		4	ERJ-2RKF1002X	$0.04
Crystal_GND24		1	ABM12W-24.0000MHZ-8-D1X-T3	$1.05
32.768 kHz		1	ABS07-32.768KHZ-T	$0.91
			total	$22.50

Figure S14. Forehead device (a) detailed block diagram, (b) component layout, and (c) bill of materials. Active components are off-the-shelf components. Passive components are in the 0402 or 0603 sizing package. The total cost of the forehead device is $22.50.

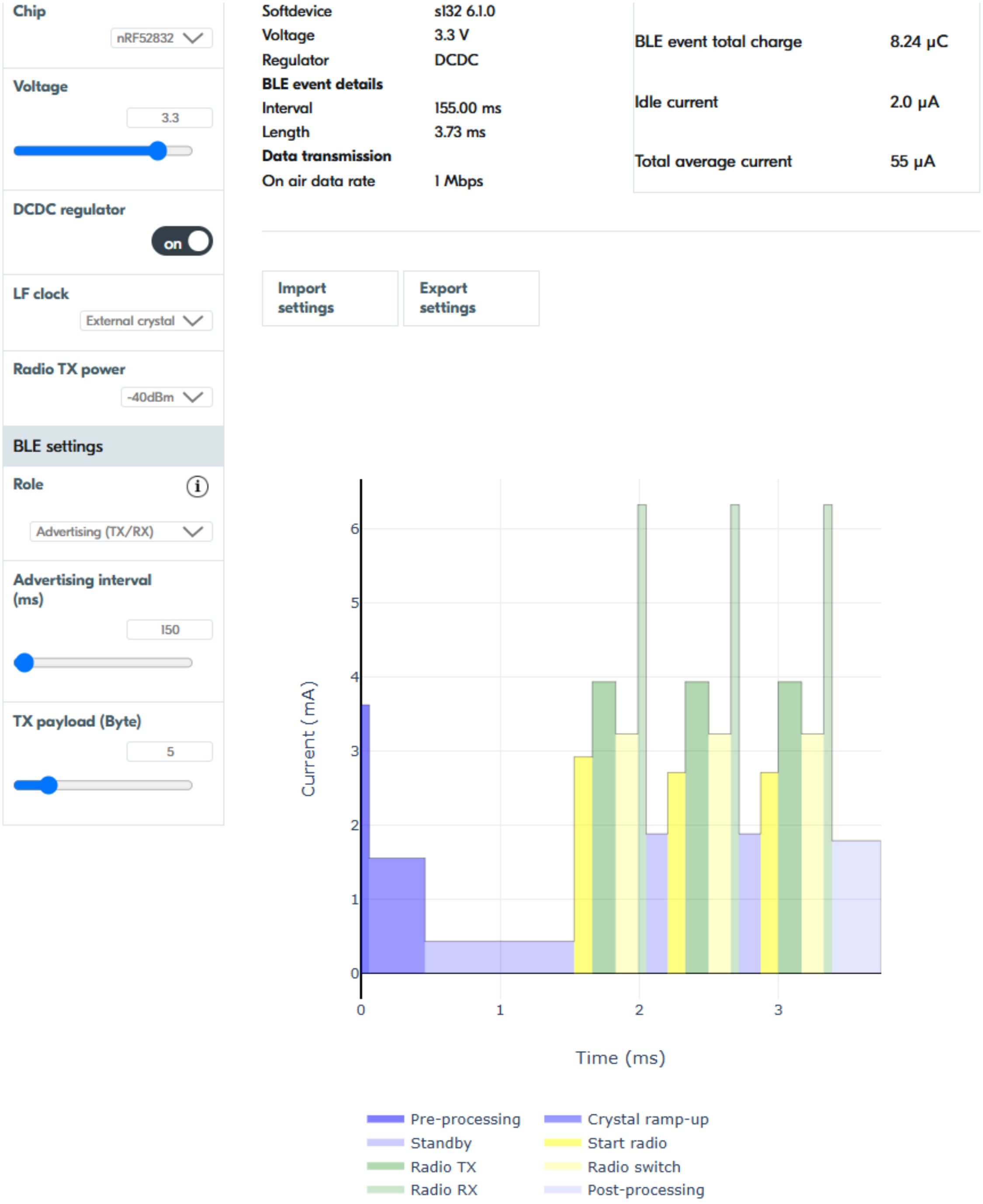

Figure S15. Power profiling simulation of the Bluetooth Low Energy (BLE) advertising event using the nRF52832 chip. Parameters were set at 3.3 V supply voltage, DCDC regulator enabled, advertising interval of 150 ms, and a payload of 5 bytes. The current profile shows BLE operational phases, including pre-processing, crystal ramp-up, radio transmission (TX), radio receiving (RX), and post-processing stages. Average current consumption during idle and active phases was 2.0 μA and 55 μA, respectively.

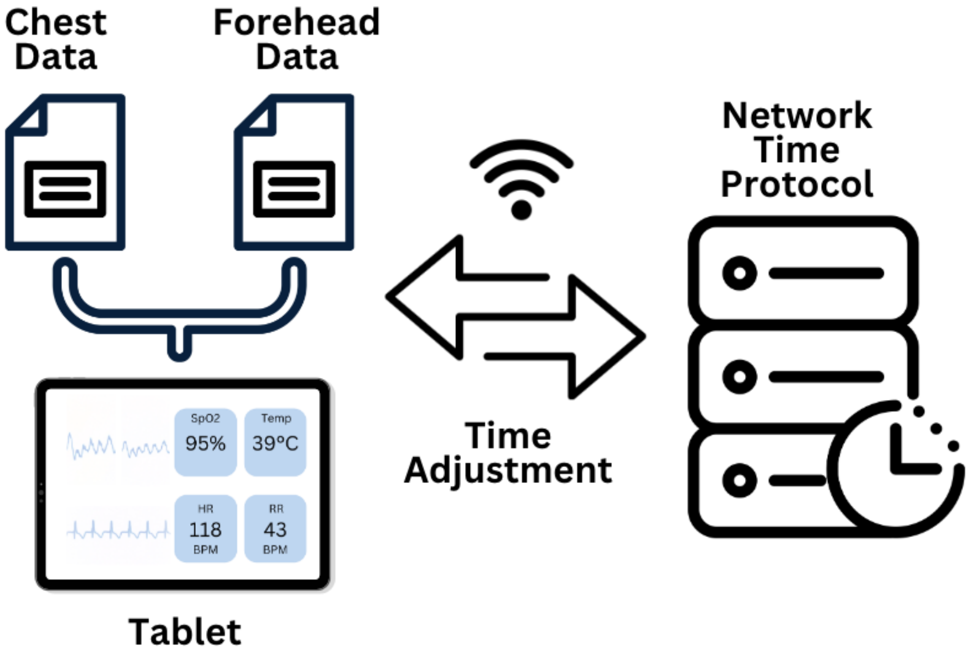

Figure S16. Time synchronization block diagram. Data packets from each Bluetooth device are sent to the paired tablet, which uses Wi-Fi and network time protocol to calibrate local clocks for data time synchronization.

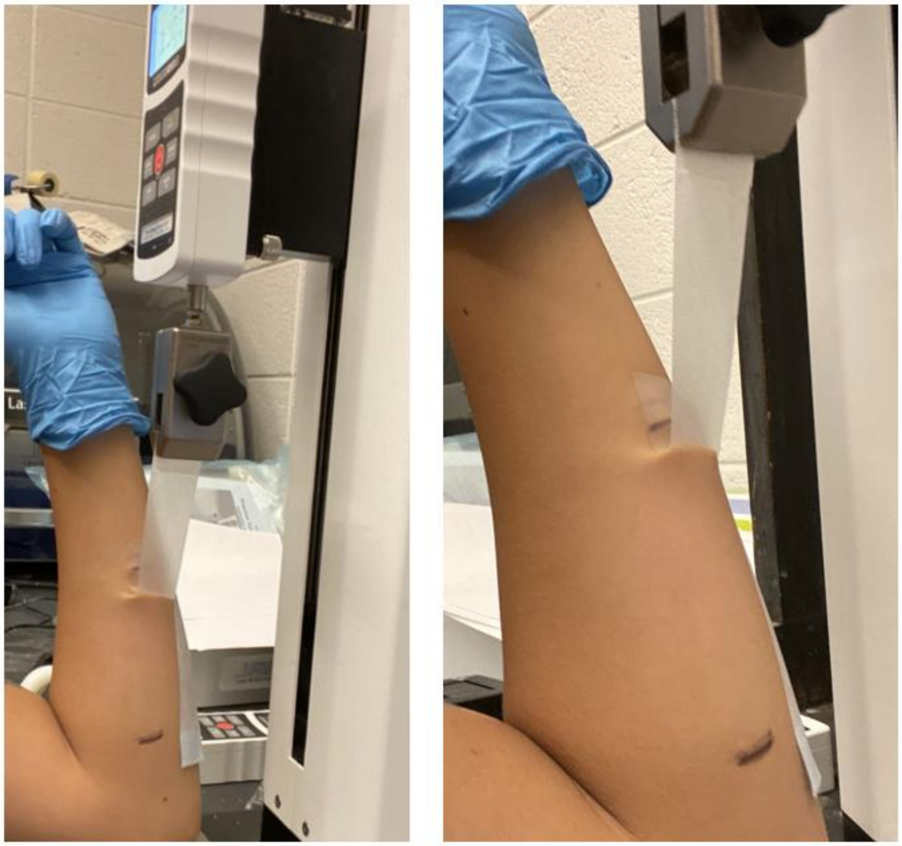

Figure S17. An experimental testing setup for chest device adhesive was done using a 3.8 cm by 10.2 cm substrate.

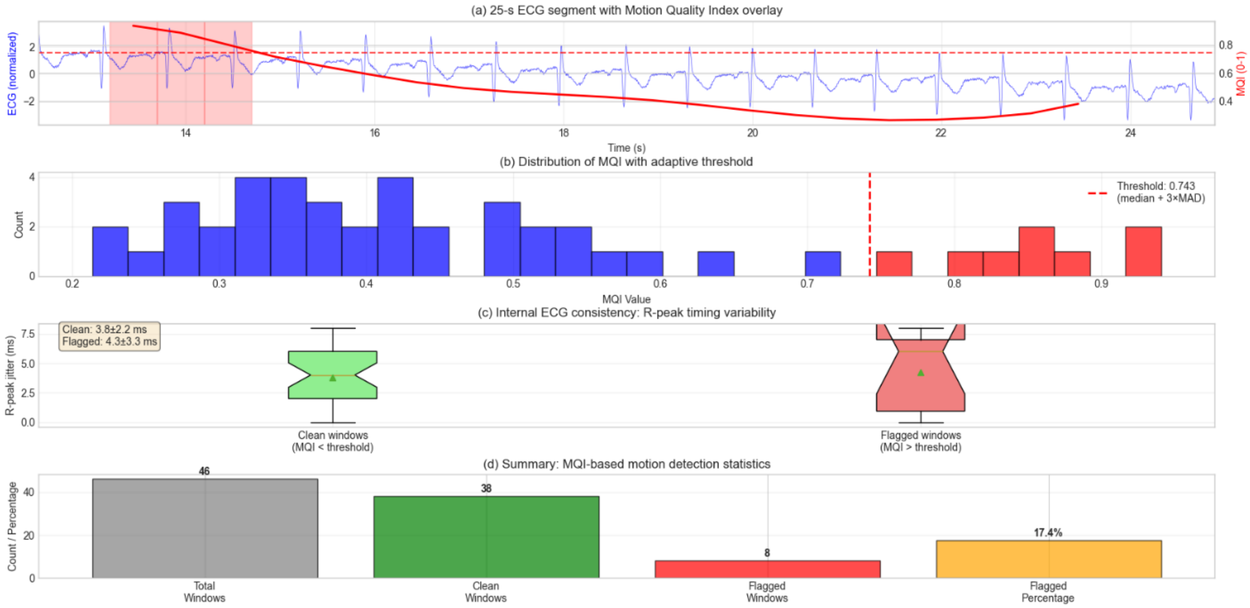

Figure S18. Software-only motion handling from ECG data

(a) 25-s ECG segment (250 Hz) with MQI (0–1) overlaid. The dashed line marks the MQI threshold; shaded regions are MQI-flagged high-motion windows (window = 2 s; hop = 0.5 s).

(b) Distribution of MQI for this recording with the same threshold indicated.

(c) Internal ECG consistency: R-peak timing jitter (ms, window-level) for non-flagged vs. flagged windows shown as median; non-parametric comparison is reported for descriptive purposes (windows overlap).

(d) Window summary for this segment: total windows, non-flagged windows, flagged windows, and flagged percentage.

Table S1: Example of a digitized version of patient logs collected periodically from nursing staff. Vitals are collected once per hour, which differs from our proposed system.

Participant ID	Date	time	Data Collector	Temperature		Heart Rate		Blood Oxygen		Respiration Rate	
605				Thermometer	GT-Biopatch	Pulse Ox	GT-Biopatch	Pulso Ox	GT-Biopatch	Count	GT-Biopatch
		4:22	Intern	34.7	34	124	161	99	94	52	49
		5:26	Intern	36.4	36	118	123	99	100	42	46
		6:25	Intern	37.2	36	138	147	94	93	60	58
		7:26	Intern	36.1	36	116	111	94	97	42	40
		8:30	Intern	37	35	122	132	98	94	46	43
		9:38	Intern	36.6	36	126	128	96	95	48	46
		10:30	Intern	36.5	35	140	116	97	100	40	44
606											
		10:40	Intern	36.3	36	128	92	97	93	50	46
		11:33	Intern	36.7	36	130	104	93	94	37	40
		12:30	Intern	36.9	36	88	112	93	91	42	47
		1:27	Intern	36.2\	37	105	120	94	84	46	40
		2:28	Intern	36.7	37	114	94	97	92	40	45
		3:27	Intern	37	36	120	99	98	89	42	43
		4:23	Intern	36.3	36	120	119	100	98	46	43
607											
		6:47	Intern	36.6	35	118	116	99	86	40	44
		7:58	Intern	36.2	34	126	139	86	95	50	36
		9:00	Intern	26.5	34	132	104	99	99	55	44
		10:14	Intern	36.3	33	138	125	99	84	40	27
		11:18	Intern	36.9	34	128	186	99	92	38	45
		12:22	Intern	36.7	35	134	122	99	96	44	52
		1:27	Intern	36.8	32	124	180	90	95	56	56
619											
			intern	36.5	35	130	114	99	98	44	38
			intern	37	36	143	139	90	95	40	37
			intern	37.5	36	133	137	89	90	41	39
			intern	37.2	35	130	133	91	94	43	40
			intern	37.4	36	123	127	93	89	42	44
			intern	37	35	120	133	96	92	40	42
			intern	38	37	126	136	92	91	38	40
			intern	37.2	37	117	127	95	96	42	39
			intern	37.4	36	125	135	97	93	41	44
			intern	37.1	35	121	139	93	96	43	46
620											
			intern	36.7	37	113	125	86	90	52	50
			intern	36.5	36	110	119	88	87	55	47
			intern	36.2	36	104	13	84	89	56	40
			intern	36.5	36	110	117	86	89	50	49
			intern	37.7	37	122	126	87	91	49	46
			intern	37.2	36	127	140	90	96	47	48
			intern	36.3	35	117	137	90	94	44	47
			intern	36.6	37	110	130	88	89	49	47
			intern	36.7	37	119	131	86	84	52	56
			intern	35.9	34	125	140	87	90	54	59
			intern	35.5	33	110	127	92	95	46	52

Video S1. Real-time operation of the wearable system.

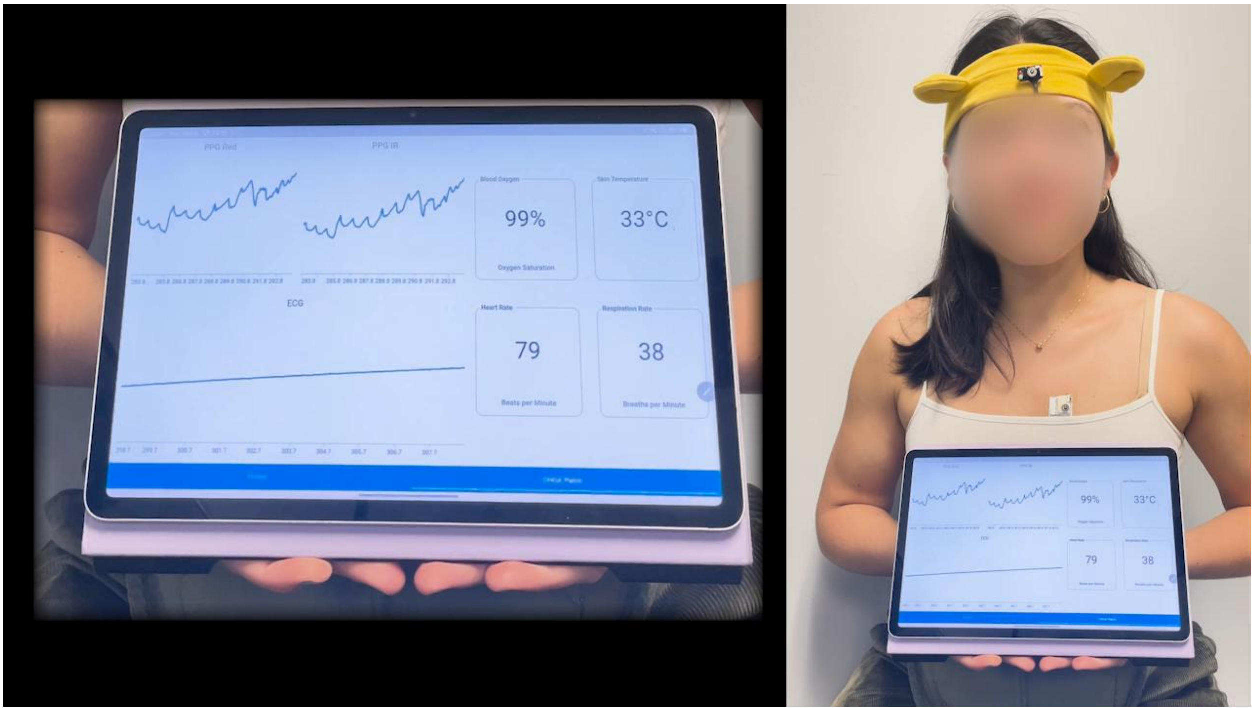

Supplement: Supplementary file 1 — 2. Rev2_SI [file 41746_2025_1974_MOESM1_ESM.pdf]
